# Structural and functional insights into the interaction between a PP01 phage gp38 tail fiber tip and an *Escherichia coli* OmpC receptor

**DOI:** 10.1128/mbio.02110-25

**Published:** 2026-01-09

**Authors:** Haruka Terasaki, Aleksandar Zdravković, Tatsuya Niwa, Ayaka Washizaki, Marina Kawaguchi, Tetsuro Yonesaki, Shuji Kanamaru, Yuichi Otsuka

**Affiliations:** 1 Department of Biochemistry and Molecular Biology, Graduate School of Science and Engineering, Saitama University 13032, Saitama, Japan; 2 Department of Life Science and Technology, Institute of Science Tokyo, Kanagawa, Japan; 3 Institute of Integrated Research, Institute of Science Tokyo, Kanagawa, Japan; 4 Laboratory of Phage Biologics, Graduate School of Medicine, Gifu University 12785, Gifu, Japan; 5 Department of Biological Sciences, Graduate School of Science, Osaka University 12936 , Osaka, Japan; The Hong Kong Polytechnic University, Kowloon, Hong Kong

**Keywords:** bacteriophages, tail fiber proteins, *Escherichia coli *O157, OmpC porin, protein-protein interactions, phage–host specificity

## Abstract

Bacteriophages exhibit strict host specificity, primarily determined by adsorption to bacterial surface receptors. However, the molecular basis underlying this specificity remains incompletely understood. Here, we investigate the interaction between outer membrane protein C (OmpC) of *Escherichia coli* O157 and gp38, the receptor-binding protein located at the tip of the long tail fibers of phage PP01. We determined the crystal structure of the receptor-binding domain (RBD) of gp38_PP01_ at 2.1 Å resolution. The structure reveals a lattice of poly-glycine type II helices with protruding receptor recognition loops, resembling that of gp38 from *Salmonella* phage S16. To identify interaction sites, we performed site-specific photo-crosslinking using *p*-benzoyl-L-phenylalanine (pBPA), followed by liquid chromatography–tandem mass spectrometry. Two critical contacts were identified: Gly208 in loop-D of gp38_PP01_ crosslinked to Ser225 and Pro226 in extracellular loop-5 of OmpC_O157_, and Tyr230 in loop-E of gp38_PP01_ to the Val304–Arg308 region in loop-7 of OmpC_O157_. A structural model of the gp38_PP01_–OmpC_O157_ complex was constructed using distance-constrained prediction and validated by targeted mutagenesis. Our findings demonstrate that PP01 phage specificity is governed by loop-E of gp38_PP01_ engaging a cleft formed by loops -5 and -7 of OmpC_O157_. These structural and functional insights enhance our understanding of phage–host recognition and may inform the rational design of engineered bacteriophages with altered host ranges.

**IMPORTANCE:**

Bacteriophages must precisely recognize and bind to specific molecules on the surface of their bacterial hosts to initiate infection, but the details of these interactions are often unclear. In this study, we examined how phage PP01 targets *Escherichia coli* O157. Using structural analysis of the phage tail fiber and a technique to capture contact points between the phage and a bacterial surface protein, we mapped the molecular basis of host recognition. We also developed a simple test system using a modified phage to identify which parts of the tail fiber are essential for binding. These methods can be broadly applied to other phages to better understand how they select their hosts. This work provides valuable insights and tools that could aid the design of phages with customized host specificity for therapeutic or biotechnological applications.

## INTRODUCTION

Bacteriophages (phages) exhibit strict host specificity, primarily determined by adsorption—the initial step of the phage life cycle (1). This process is mediated by interactions between phage receptor-binding proteins (RBPs), such as tail fibers, and specific receptor molecules on the bacterial surface. However, the molecular details of these interactions remain largely unresolved. Understanding these mechanisms is essential not only for elucidating phage host specificity but also for advancing phage therapy, the use of natural or engineered phages as antibacterial agents against multidrug-resistant bacteria—a growing global health threat.

Phages with contractile tails belong to the family *Myoviridae* (now reclassified as *Straboviridae*), including the well-studied T-even phages (subfamily *Tevenvirinae*), which are further divided into T4-type and T2-type groups based on tail fiber protein composition. Phage T4, a representative T4-type phage, consists of a head, a contractile tail, and a baseplate to which six long tail fibers are attached. These long tail fibers reversibly bind to receptors on the surface of *Escherichia coli* during the initial adsorption phase (2). One such receptor is outer membrane protein C (OmpC), a trimeric porin composed of 16-stranded antiparallel β-barrels, with extracellular loops from each monomer contributing to receptor recognition (3, 4). Several of these loops are critical for T4 adsorption. A T4 long tail fiber is assembled from four gene products—gp34, gp35, gp36, and gp37 (5)—with the distal rod formed by gp36 and gp37 homotrimers (6). gp38 functions as a molecular chaperone for gp37 folding and is released following assembly (7). Islam et al. (8) identified gp37 residues involved in binding OmpC and lipopolysaccharide (LPS), proposing that the trimeric gp37 tip contains three symmetrically arranged binding sites that engage dynamically with these receptors. Our previous work also identified critical residues and suggested that the gp37 trimer tip docks into the central region of trimeric OmpC, with structural complementarity at the interface being essential for docking (9).

In contrast to T4-type phages, T2-type phages possess an RBP, gp38, located at the tip of the gp37 trimer. In 1987, Henning’s group, through sequence alignment of four T2-type phages, identified that gp38 contains hypervariable regions flanked by glycine-rich motifs (10, 11). They later introduced mutations into these hypervariable regions in phage OX2, generating variants with altered receptor specificity for *E. coli* (12, 13). Studies using *Shigella* and its T2-type phages Sf22 and KRT47 identified *ompC* mutants resistant to infection, suggesting that electrostatic interactions contribute to gp38–OmpC binding (14). A structural study by Dunne et al. (15) revealed the architecture of the gp37 trimer–gp38 complex from *Salmonella* phage S16. S16 gp38 contains rare poly-glycine type II (PG_II_) helices folded into a tightly packed lattice—termed the “PG_II_ sandwich”—from which hypervariable loops protrude and are presumed to mediate host receptor binding. S16 targets extracellular loops of *Salmonella* OmpC for adsorption (15, 16). Although crystal structures S16 gp38 and *Salmonella* OmpC are available, their precise interaction interface remains unresolved. In recent years, structural prediction of protein complexes, such as AlphaFold3, has significantly advanced. However, it remains challenging to predict structures for phage RBP and bacterial Omp complex that satisfy experimental data.

Here, we determined the crystal structure of the receptor-binding domain (RBD) of gp38 from the virulent PP01 phage. This phage, isolated from swine stool samples in Japan alongside enterohemorrhagic *E. coli* O157:H7 (17), uses OmpC as its receptor (18). Sequence alignment confirmed that PP01 belongs to the T2-type group. To identify gp38 residues critical for OmpC binding, we incorporated *p*-benzoyl-L-phenylalanine (pBPA) into predicted receptor-binding loops, enabling UV cross-linking followed by liquid chromatography–tandem mass spectrometry (LC-MS/MS) analysis. Using the resulting cross-linked residue pairs as distance restraints, we constructed a structural model of the gp38–OmpC complex. Mutational analyses in both gp38 and OmpC validated the model and confirmed direct interactions. Collectively, these findings provide molecular insight into phage tail fiber–receptor recognition. This enhanced understanding may facilitate the rational design of engineered phages with altered host specificity for therapeutic applications.

## RESULTS

### Crystal structure of the RBD of gp38_PP01_ (gp38_PP01_RBD)

**Fig 1:**
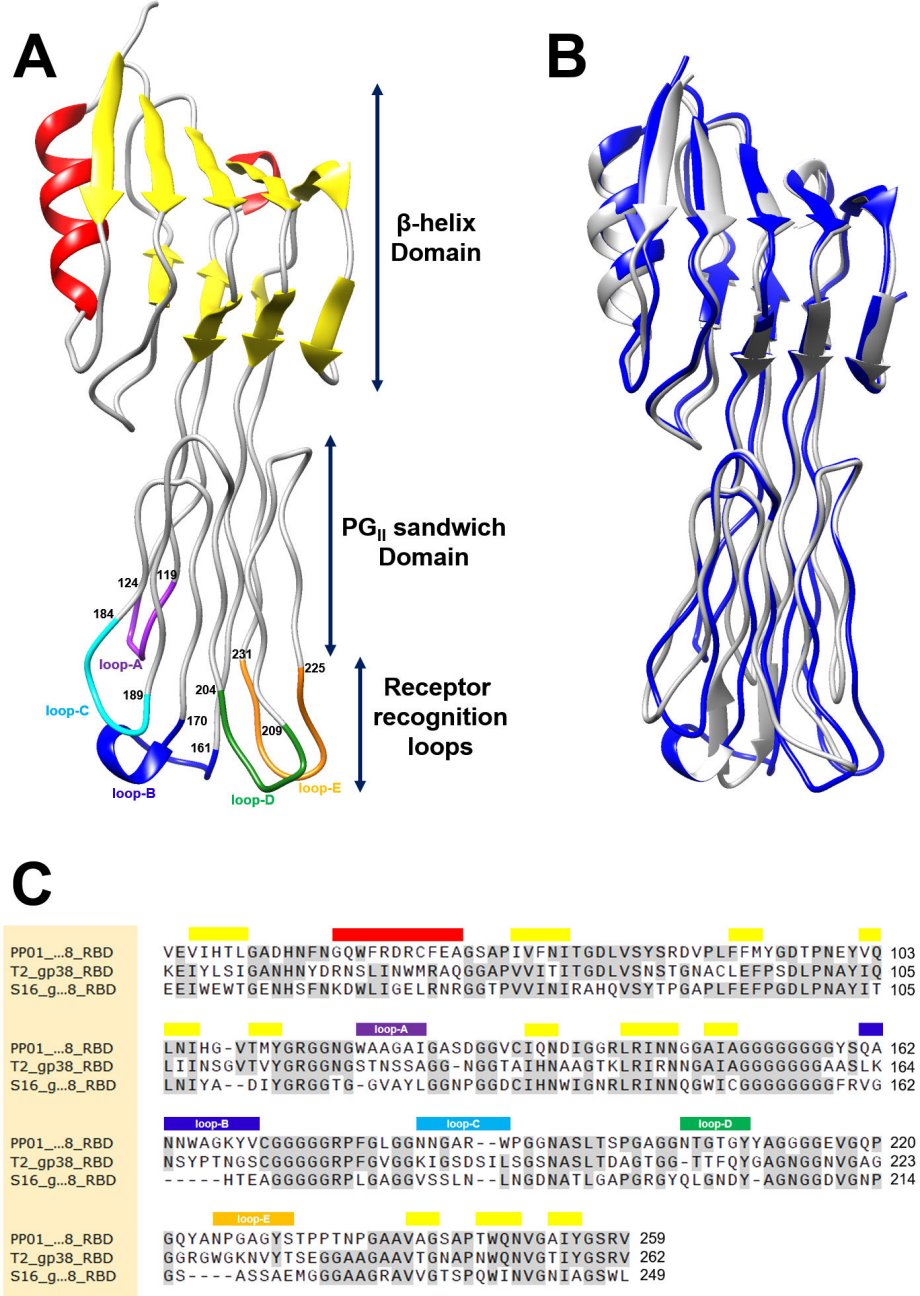
Structure of the PP01 phage gp38 RBD. (**A**) Crystal structure of the PP01 gp38 RBD. α-helices are shown in red and β-sheets in yellow. Putative receptor recognition loops A to E are colored purple, blue, cyan, green, and orange, respectively. The starting and ending residue numbers of loops are indicated. (**B**) Structural superposition of the RBDs of PP01 gp38 (blue) and S16 gp38 (gray; PDB ID: 6F45). (**C**) Sequence alignment of the gp38 RBDs from PP01, T2, and S16 phages, generated using ClustalOmega. Conserved residues are highlighted in gray. Red and yellow bars represent α-helices and β-sheets, respectively. Color-coded bars above the sequences indicate the positions of putative receptor recognition loops A–E, matching the colors used in panel (**A**).

The structure of the superfolder green fluorescent protein (sfGFP)-fused RBD of gp38_PP01_ was determined by molecular replacement, using the coordinates of sfGFP (PDB ID: 4J8A) and the RBD of phage S16 gp38 (PDB ID: 6F45) as search models. Crystals diffracted to 2.1 Å resolution and belonged to space group P4_3_2_1_2 (Table 1). Although two sfGFP-gp38_PP01_RBD fusion molecules were present in the asymmetric unit, electron density corresponding to one sfGFP molecule was not observed (Fig. S1). The two gp38_PP01_RBD molecules present in the asymmetric unit adopted nearly identical conformations, with a root-mean-square deviation (RMSD) of 0.143 Å for all 218 Cα atoms. The overall structure of gp38_PP01_RBD (Fig. 1A) comprises a β-helix domain, a poly-glycine II (PG_II_) sandwich domain, and receptor recognition loops. Except for the hypervariable region, the structure closely resembles that of the S16 gp38RBD (RMSD 1.43 Å for 202 equivalent Cα atoms) (Fig. 1B), consistent with their primary sequence similarity (Fig. 1C). The putative receptor-binding loops were identified as loop-A (W119–I124), loop-B (Q161–V170), loop-C (N184–W189), loop-D (N204–Y209), and loop-E (N225–S231).

**TABLE 1:** X-ray data collection, processing, and refinement statistics for the crystal structure of sfGFP fused C-terminal RBD structure of gp38_PP01_

Statistic	Result for sfGFP-gp38C^41–259^
Data collection	
Wavelength (Å)	1.000
Resolution range (Å)	36.17–2.1 (2.175–2.1)^*a*^
Space group	*P*4_3_2_1_2
Unit cell (Å)	a=172.217, b=172.217, c=79.71
Total reflections	1,849,930 (183,784)
Unique reflections	70,176 (6,886)
Multiplicity	26.4 (26.7)
Completeness (%)	99.96 (100.00)
Mean I/σ(I)	23.19 (1.98)
Wilson B-factor (Å^2^)	36.73
*R*_merge^*b*^_	0.1463 (1.854)
Refinement	
Reflections used in refinement	70,169 (6,887)
Reflections used for *R*_free_	2,130 (206)
*R*_work^*c*^_	0.1754 (0.2609)
*R*_free_	0.2076 (0.3344)
CC (work)	0.960 (0.827)
CC (free)	0.945 (0.670)
Number of non-hydrogen atoms	5,496
Proteins	4,881
Ligands	24
Solvent	591
Protein residues	666
RMS (bonds)	0.013
RMS (angles)	1.31
Ramachandran favored (%)	97.42
Ramachandran allowed (%)	2.58
Ramachandran outliers (%)	0
Rotamer outliers (%)	0.62
Clashscore	2.73
Average B-factor (Å^2^)	42.59
Proteins	41.65
Ligands	41.88
Solvent	50.41
PDB accession code	9V85

^*a*^ Values in parentheses are for the highest resolution bin (where applicable).

^*b*^ *R*_merge_ = Σ_h_Σ_i_|I_hi_ − |/Σ_h_Σ_i_|I_hi_|, where I_hi_ is the intensity of the ith measurement of the same reflection and is the mean observed intensity for that reflection.

^*c*^ *R*_work_ = Σ||F_obs_(hkl)| − | F_calc_(hkl)||/Σ| F_obs_(hkl)|. R_free_ is the cross-validation R_work_ for the test set (3%) of reflections omitted in model refinement. RMS, root mean square.

### Photo-cross-linking analysis between gp38_PP01_RBD and OmpC_O157_

**Fig 2:**
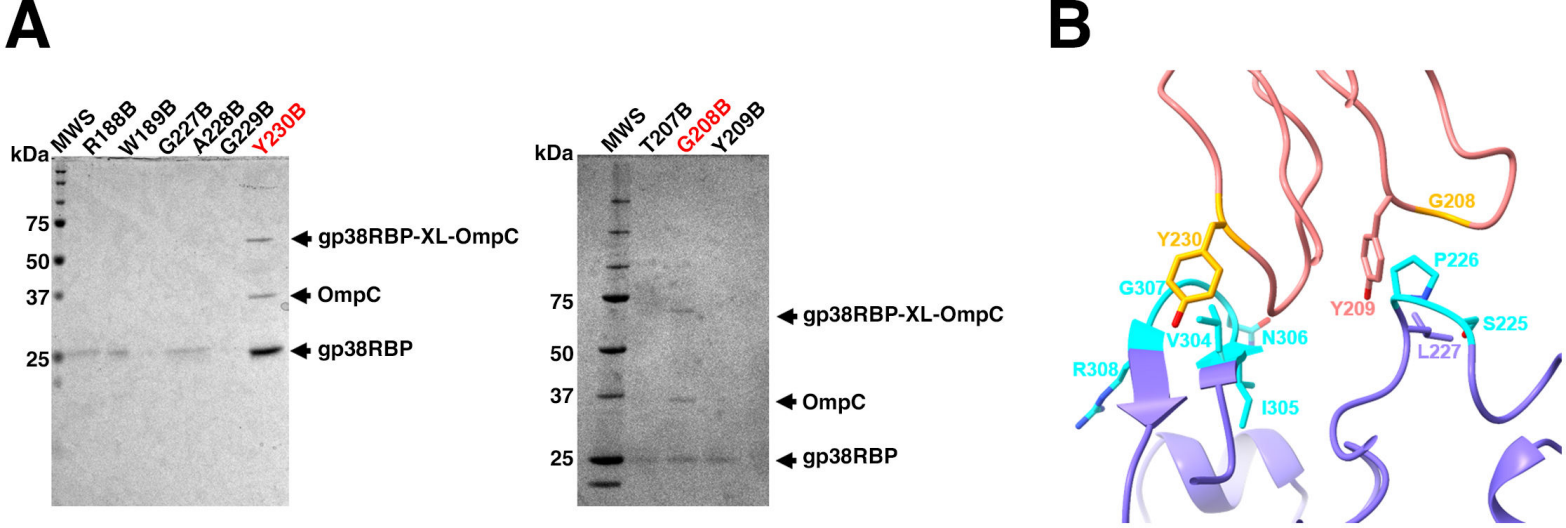
UV cross-linking analysis of gp38_PP01_ and OmpC_O157_. (**A**) SDS-PAGE analysis of Ni-NTA-bound solubilized membrane fractions after UV cross-linking. Cross-linked mutants show three bands: the gp38–OmpC complex (gp38RBD–XL–OmpC), unlinked OmpC, and free gp38RBD. (**B**) Cross-linked residue pairs identified by LC-MS/MS were mapped onto structural models. gp38 is shown as a pink ribbon and OmpC as a slate blue ribbon. Cross-linked residues in gp38 and OmpC are labeled in orange and cyan, respectively.

Although the crystal structure of gp38_PP01_RBD was solved, it did not reveal specific interaction residues with OmpC_O157_. To identify contact sites, 19 unique amber codon mutations, each substituting a native codon with the UAG stop codon, were introduced into the loop region of a His-tagged gp38_PP01_RBD expression vector. These constructs, along with a plasmid encoding an amber suppressor tRNA specific for *p*-benzoyl-L-phenylalanine (pBPA), were used to express pBPA-labeled variants of gp38_PP01_RBD in *E. coli* cultured with pBPA supplementation. Clarified lysates from each variant were mixed with the membrane fraction of *E. coli* O157 and subjected to UV irradiation. As shown in Fig. 2A, two out of the 19 mutants— G208B and Y230B (B denotes incorporation of pBPA)—cross-linked with OmpC. Gly208 is located in loop-D and Tyr230 in loop-E of gp38_PP01_RBD. Sodium dodecyl sulfate–polyacrylamide gel electrophoresis (SDS-PAGE) revealed a 38.5 kDa band corresponding to free OmpC, along with bands at 23.6 kDa (His-tagged [his]-gp38_PP01_RBD) and 62.1 kDa (cross-linked his-gp38_PP01_RBD-XL-OmpC_O157_ complex). The estimated molar ratio of OmpC_O157_ to his-gp38_PP01_RBD-XL-OmpC_O157_ complex was approximately 2:1. Since the irradiated membrane fraction was solubilized by SDS solution and pulled down by Ni-NTA with the his-tag on his-gp38_PP01_RBD, suggesting that free OmpC binds to cross-linked OmpC. Thus, OmpC forms a trimer in 1% SDS, with a single gp38_PP01_RBD molecule cross-linked to one monomer within the OmpC_O157_ trimer.

### Cross-linked residues identified by LC-MS/MS

The his-gp38_PP01_RBD-XL-OmpC_O157_ cross-linked band excised from the SDS-PAGE gel was digested with either trypsin and endoproteinase GluC or trypsin and ProAlanase. The resulting peptides were analyzed by LC-MS/MS, and cross-linked residues were identified using pLink software. For the G208B mutant, cross-linking was detected with Ser225 and Pro226 of OmpC_O157_ (Fig. S2A; Table 2), both located within extracellular loop-5. The hydrophobic nature of Pro226 and the adjacent Leu227 likely contributes to hydrophobic interactions with Tyr209 of gp38_PP01_, which is adjacent to Gly208 (Fig. 2B). For the Y230B mutant, cross-linking occurred with OmpC_O157_ residues spanning Val304 to Arg308 (Fig. S2B; Table 3), located in extracellular loop-7. Among these, Val304 is a strong candidate for hydrophobic interaction with Tyr230 of gp38_PP01_ (Fig. 2B).

**TABLE 2:** Cross-linking mass spectrometry results for gp38_PP01_-G208Bpa identified using pLink2

Detected cross-linked peptide sequence^*a*^	Linkage positions	Summed number of CSMs^*b*^
$\;\begin{array}{c} WPGGNASLTSPGAGGNTGT\underline{\text{B}}YYAGGGGE\\ \;\;\;\;\;\;|\\ \;\;\;\;\;\;TDDQNS\underline{\text{P}}LYIGNGDR \end{array}$	gp38 208 (Bpa) and OmpC 226 (Pro)	12
$\;\begin{array}{c} \;WPGGNASLTSPGAGGNTGT\underline{\text{B}}YYAGGGGE\\ \;\;\;\;\;\;|\\ \;\;\;\;\;TDDQN\underline{\text{S}}PLYIGNGD \end{array}$	gp38 208 (Bpa) and OmpC 225 (Ser)	4

^*a*^ “B” represents pBPA.

^*b*^ Sum of the number of CSMs for six measurements in three replicate samples. Deamidated peptides were included. Only cross-link sites with a summed number of CSM value more than two are shown.

**TABLE 3:** Cross-linking mass spectrometry results for gp38_PP01_-Y230Bpa identified using pLink2

Detected cross-linked peptide sequence^*a*^	Linkage positions	Summed number of CSMs^*b*^
$\;\begin{array}{c} \;GAG\underline{\text{B}}STPP\\ |\\ \;\;\;NLGVIN\underline{\text{G}}RNYDDEDILK \end{array}$	gp38 230 (Bpa)– OmpC 307 (Gly)	12
$\;\begin{array}{c} \;\;GAG\underline{\text{B}}STPP\\ |\\ \;\;\;NLGV\underline{\text{I}}NGRNYDDEDILK \end{array}$	gp38 230 (Bpa)– OmpC 305 (Ile)	9
$\;\begin{array}{c} \;GAG\underline{\text{B}}STPP\\ |\\ \;\;NLGVING\underline{\text{R}}RNYDDEDILK \end{array}$	gp38 230 (Bpa)– OmpC 308 (Arg)	9
$\;\begin{array}{c} NPGAG\underline{\text{B}}STPP\\ \;|\\ \;\;\;\;NLGVING\underline{\text{R}}NYDDEDILK \end{array}$	gp38 230 (Bpa)– OmpC 308 (Arg)	6
$\;\begin{array}{c} \;\;GAG\underline{\text{B}}STPPTNP\\ |\\ \;\;NLGVIN\underline{\text{G}}RNYDDEDILK \end{array}$	gp38 230 (Bpa)– OmpC 307 (Gly)	5
$\;\begin{array}{c} GAG\underline{\text{B}}STP\\ |\\ \;\;\;DAG\underline{\text{I}}NTDNIVA \end{array}$	gp38 230 (Bpa)– OmpC 353 (Ile)	5
$\;\begin{array}{c} \;\;GAG\underline{\text{B}}STPPTNP\\ |\\ \;\;\;NLGV\underline{\text{I}}NGRNYDDEDILK \end{array}$	gp38 230 (Bpa)– OmpC 305 (Ile)	4
$\;\begin{array}{c} GAG\underline{\text{B}}STP\\ |\\ \;\;\;NLGVIN\underline{\text{G}}GRNYDDEDILK \end{array}$	gp38 230 (Bpa)– OmpC 307 (Gly)	4
$\;\begin{array}{c} \;GAG\underline{\text{B}}STPP\\ |\\ \;\;\;NLGVI\underline{\text{N}}GRNYDDEDILK \end{array}$	gp38 230 (Bpa)– OmpC 306 (Asn)	3
$\;\begin{array}{c} \;GAG\underline{\text{B}}STPPTNP\\ |\\ \;NLGVI\underline{\text{N}}GRNYDDEDILK \end{array}$	gp38 230 (Bpa)– OmpC 306 (Asn)	3

^*a*^ “B” represents pBPA.

^*b*^ Sum of the number of CSMs for two measurements in one sample. Deamidated peptides were included. Only cross-link sites with a summed number of CSM value more than two are shown.

### Complex models based on cross-linking analysis

**Fig 3:**
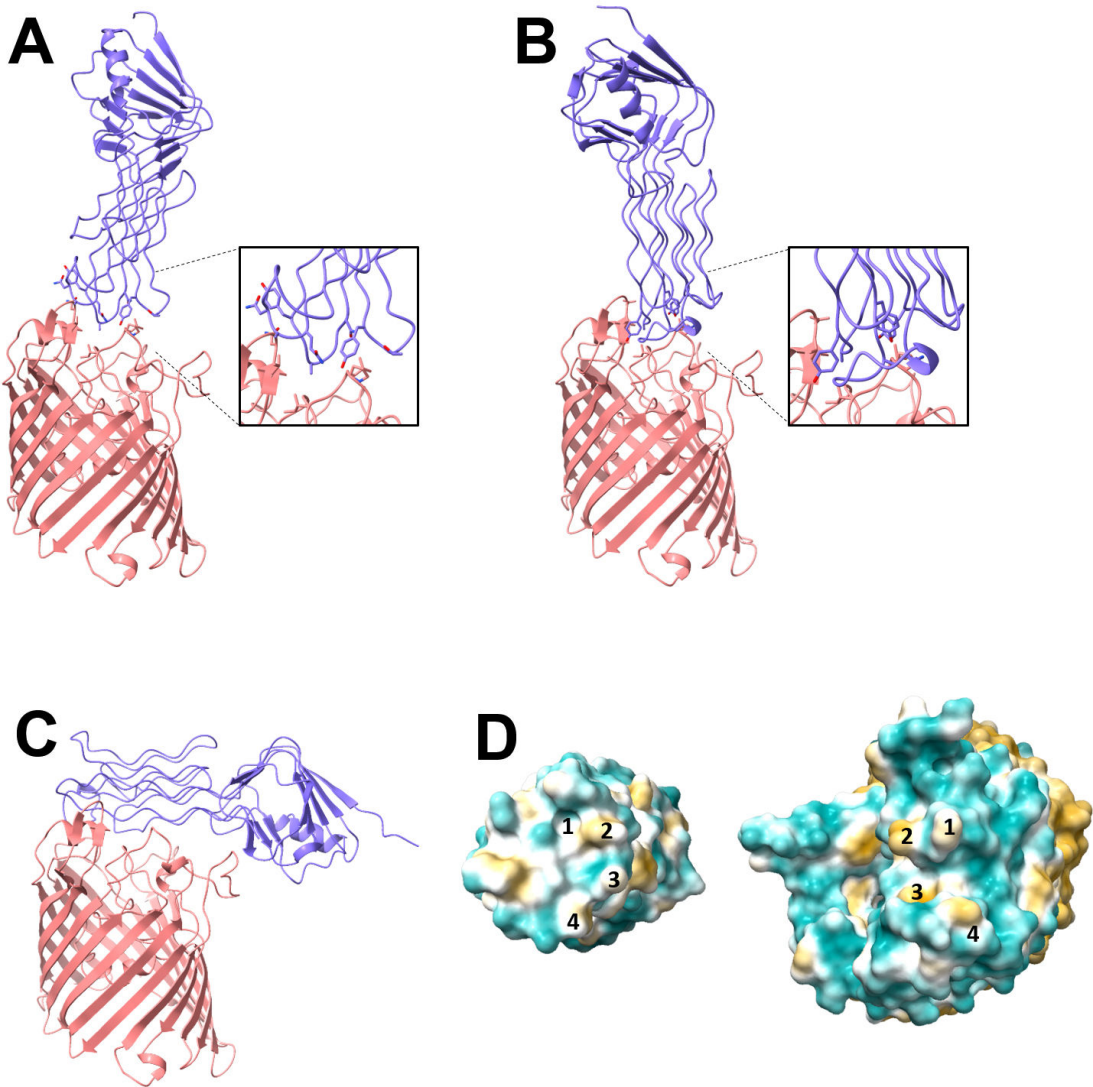
Predicted structure of the gp38_PP01_-OmpC_O157_ complex. (**A**) Manually fitted complex structure based on UV cross-linking results. (**B**) Predicted complex structure of gp38_PP01RBD_ and OmpC_O157_ using Chai-1 with distance restraints. (**C**) Predicted complex structure of gp38_PP01RBD_ and OmpC_O157_ using AlphaFold3. OmpC_O157_ and gp38_PP01_RBD are shown as a ribbon model (slate blue and salmon pink, respectively). (**D**) Hydrophobic surface representations of gp38_PP01RBD_ (bottom view, left) and the AlphaFold3-predicted OmpC_O157_ monomer (top view, right). Surface hydrophobicity is rendered from cyan (hydrophilic) to yellow (hydrophobic). Numbers indicate “hill” and “valley” regions contributing to surface complementarity.

The crystal structure of gp38_PP01_RBD and the AlphaFold-predicted structure of OmpC_O157_ (AF-Q8XE41-F1-v4; AlphaFold Protein Structure Database) (19, 20) were used to manually construct a gp38_PP01_RBD–OmpC_O157_ complex model informed by cross-linking data (Tables 2 and 3) (Fig. 3A). A second model was generated using the Chai-1 structure prediction server, which allows the incorporation of distance constraints between cross-linked residues (Fig. 3B). Both models revealed similar interaction interfaces, with the Chai-1 model providing closer contacts via conformational adjustments that minimized steric clashes. It is to be noted that AlphaFold3 is not able to predict the complex models which satisfy the cross-linking data (Fig. 3C; Fig. S3). The manually constructed and the Chai-1 models suggest that gp38_PP01_ recognizes OmpC_O157_ primarily through the extracellular surface geometry and hydrophobic interactions. As shown in Fig. 3D, hydrophobic “hills” (#1 and #4) and “valleys” (#2 and #3) of OmpC_O157_ are complemented by corresponding hydrophobic “valleys” (#1 and #4) and “hills” (#2 and #3) on gp38_PP01_, forming a “key-and-keyhole” interface. Based on these models, the residues likely involved in complex formation are located in loops -D and -E of gp38_PP01_ and loops -7 and -5 of OmpC_O157_.

### Necessity of OmpC_O157_ loops for PP01 adsorption

**Fig 4:**
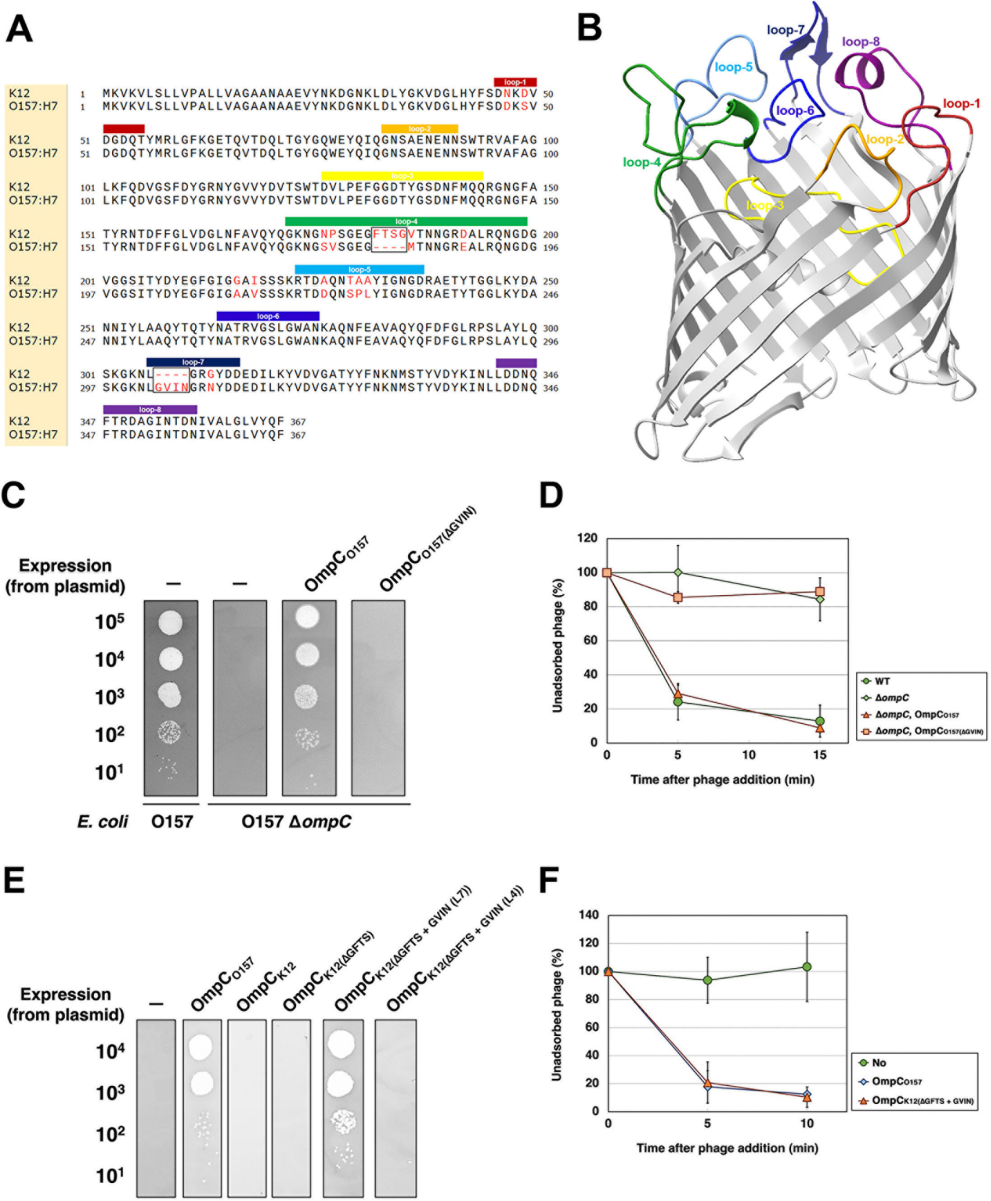
Requirement of the GVIN sequence in OmpC_O157_ loop-7 for PP01 adsorption. (**A**) Sequence alignment of OmpC from *E. coli* strains K12 and O157 generated using ClustalOmega. Non-conserved residues are colored in red. Color-coded bars above the sequences indicate the positions of extracellular loops 1–8, which are colored brown, orange, yellow, green, sky blue, blue, dark blue, and purple, respectively. Black boxes highlight strain-specific insertions. (**B**) AlphaFold3 predicted structure of OmpC_O157_. The colored residues indicate the extracellular loops 1–8, matching the colors used in panel (**A**). (**C**) Growth of PP01 phage on cells expressing OmpC_O157_ mutants. Serially diluted phage suspensions were spotted onto LB plates containing 0.1% L-Arabinose (L-ara) seeded with wild-type (WT) O157 or ∆*ompC_O157_* cells harboring plasmids expressing WT or GVIN-deleted OmpC_O157_. “–” denotes no plasmid. (**D**) Adsorption of PP01 phage on cells expressing OmpC_O157(ΔGVIN)_. The fraction of unadsorbed phages was determined as described in Materials and Methods. Symbols: ●, WT O157; ◆, Δ*ompC_O157_*; ▲, Δ*ompC_O15_*_7_ + pBAD33-OmpC_O157_; ■, Δ*ompC_O157_* + pBAD33-OmpC_O157(ΔGVIN)_. (**E**) Growth of PP01 phage on cells expressing various OmpC_K12_ mutants. Phage suspensions were spotted onto LB plates containing 0.1% L-ara seeded with Δ*ompC_O157_* cells harboring plasmids expressing OmpC_O157_, OmpC_K12_, or OmpC_K12_ mutants. “–” denotes no plasmid. (**F**) Adsorption of PP01 phage on cells expressing various OmpC_K12_ mutants. Symbols: ●, Δ*ompC_O157_* + pBAD33 (empty vector); ✦, Δ*ompC_O157_* + pBAD33-OmpC_O157_; ▲, Δ*ompC_O157_* + pBAD33-OmpC_K12(ΔGFTS + GVIN)_.

To validate the predicted gp38–OmpC complex structures, we examined the insertion of four residues—Gly303, Val304, Ile305, and Asn306 (GVIN)—in loop-7 of OmpC_O157_ (Fig. 4A and B). These residues are absent in OmpC_K12_, and PP01 cannot adsorb to OmpC_K12_ (17), suggesting a potential role in host-specific recognition. Cross-linking analysis indicated that Tyr230 of gp38_PP01_ lies near the Ile305–Gly307 region of OmpC_O157_.

To test the functional importance of GVIN, we constructed an OmpC_O157∆GVIN_ mutant in which the GVIN residues were deleted and expressed it in *E. coli* O157 lacking endogenous *ompC* (∆*ompC_O157_*). The OmpC_O157∆GVIN_ mutant was present in the membrane fraction at levels comparable to WT (Fig. S4A). However, no plaques were formed on ∆*ompC_O157_* cells expressing OmpC_O157∆GVIN_, even when 10^5^ PFU were spotted (Fig. 4C), and adsorption of PP01 to these cells was nearly absent (Fig. 4D).

Next, we assessed whether PP01 could adsorb to a chimeric OmpC_K12_, in which loop-7 was replaced by that of OmpC_O157_. To exclude the influence of loop-4, we first deleted the GFTS motif (specific to OmpC_K12_, Gly181 to Ser184) to generate OmpC_K12∆GFTS_. Spot tests confirmed that this deletion did not cause PP01 to fail to adsorb to OmpC_K12_ (Fig. 4E). In cells expressing OmpC_K12ΔGFTS+GVIN(L7)_—bearing the GVIN insertion into loop-7—PP01 formed plaques with an efficiency of plating (EOP) equivalent to that on WT OmpC_O157_, and efficient adsorption was observed (Fig. 4F). The chimeric OmpC_K12ΔGFTS+GVIN(L7)_ was also detected in the membrane fraction (Fig. S4B). In contrast, no plaques formed on cells expressing the chimeric OmpC_K12∆GFTS+GVIN(L4)_, in which the GFTS sequence in loop-4 of OmpC_K12_ was replaced with GVIN. These results indicate that the GVIN motif in loop-7 is essential for PP01 adsorption.

**Fig 5:**
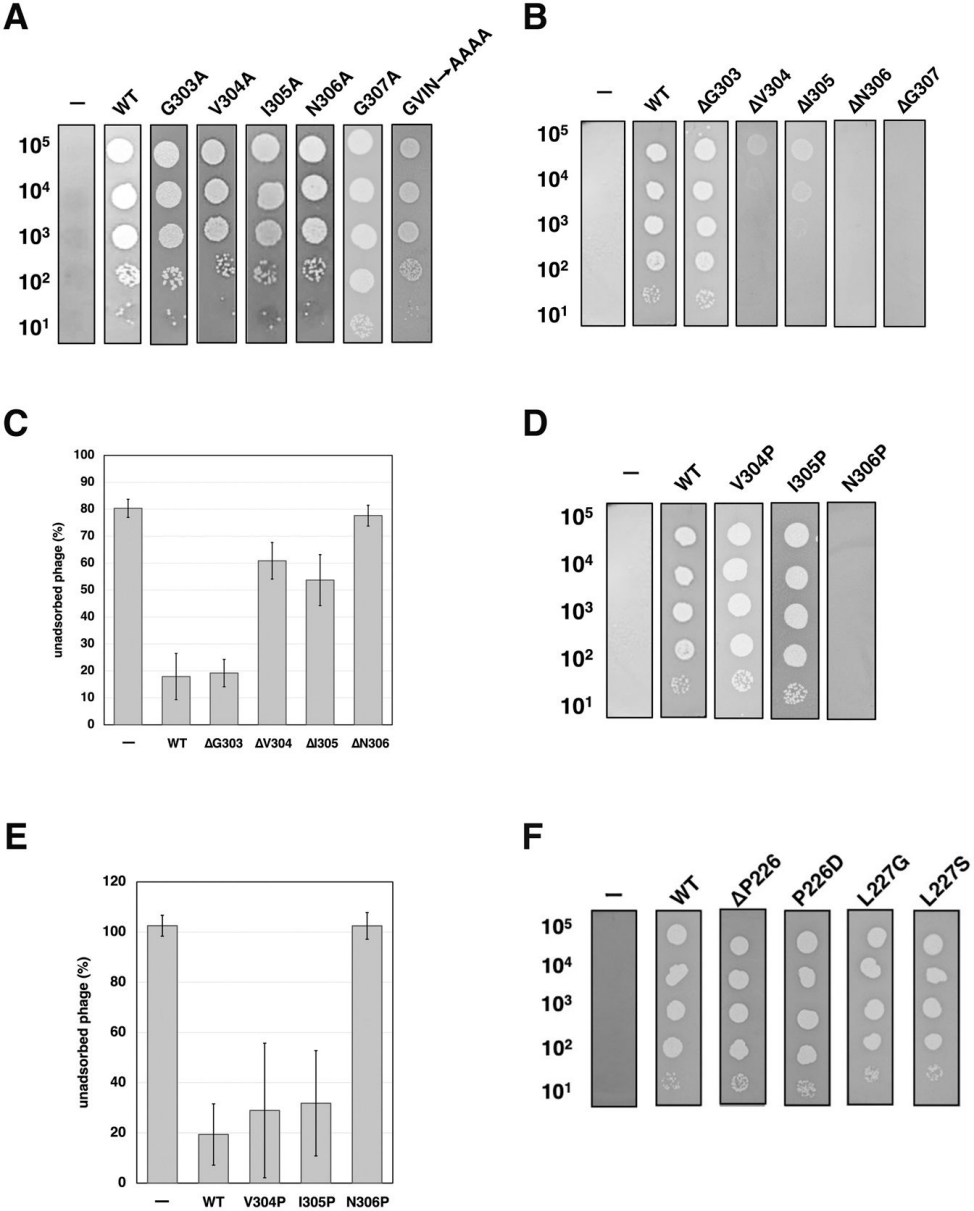
Importance of the GVING motif in OmpC_O157_ loop-7 for PP01 adsorption. (**A, B, D, F**) Growth of PP01 phage on ∆*ompC_O157_* cells expressing various OmpC_O157_ loop-7 mutants. Serially diluted phage suspensions were spotted onto LB plates containing 0.1% L-arabinose (L-ara), seeded with ∆*ompC_O157_* cells harboring plasmids expressing WT or mutant OmpC_O157_ proteins. “–” indicates no plasmid. (**C, E**) Adsorption of PP01 phage on ∆*ompC_O157_* cells expressing OmpC_O157_ loop-7 mutants. The fraction of unadsorbed phages was determined 10 min after phage addition using WT O157 as the indicator strain. “–” indicates no plasmid.

To dissect the individual contributions of these residues, each amino acid in the GVIN motif was substituted with alanine. Surprisingly, PP01 formed plaques on cells expressing each of the Ala-substituted OmpC variants (Fig. 5A). However, upon deletion of individual residues, PP01 failed to infect cells expressing OmpC_O157∆N306_ or OmpC_O157∆G307_ (Fig. 5B), while it formed turbid plaques with significantly reduced EOP on cells expressing OmpC_O157∆V304_ or OmpC_O157∆I305_. Correspondingly, adsorption was severely impaired in the ΔN306 and ΔG307 mutants and moderately reduced in the ΔV304 and ΔI305 mutants (Fig. 5C).

Proline substitutions were next introduced into each position. PP01 formed plaques on cells expressing OmpC_O157V304P_ and OmpC_O157I305P_, but not on those expressing OmpC_O157N306P_ (Fig. 5D). Adsorption of PP01 to cells expressing OmpC_O157N306P_ was negligible (Fig. 5E), suggesting that gp38_PP01_ may recognize the main chain conformation of the Val304–Gly307 region.

Cross-linking analysis also revealed an interaction between loop-D (Gly208) of gp38_PP01_ and loop-5 (Pro226 and Leu227) of OmpC_O157_. However, PP01 was able to infect cells expressing a chimeric OmpC_K12_ in which only the GVIN motif was inserted into loop-7 (Fig. 4E), suggesting that sequence specificity in loop-5 is less critical. Indeed, PP01 infection was not affected by the P226D substitution or P226 deletion (Fig. 5F), and the L227G and L227S mutants remained permissive to PP01 infection. Compared to loop-7, which forms a rigid anti-parallel β-sheet, loop-5 appears structurally flexible, and single-residue deletions or mutations are less likely to disrupt gp38_PP01_ recognition.

### Construction of T4 phage mutants with chimeric long tail fibers

**Fig 6:**
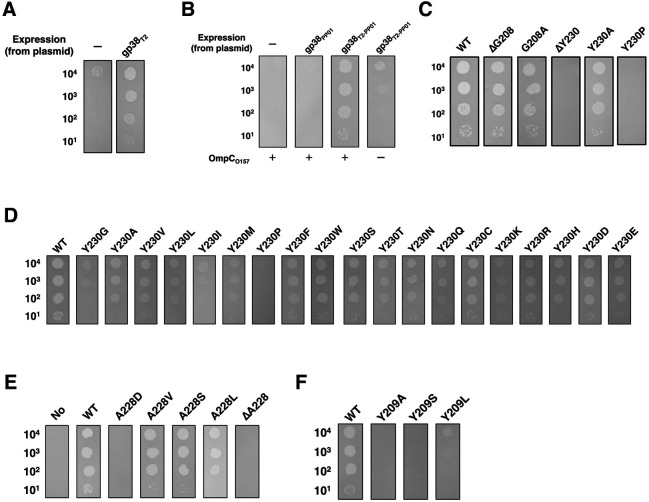
Functional contribution of gp38_PP01_ loops to adsorption onto OmpC_O157_. (**A**) Growth of DT2(∆g38) phage on cells expressing T2 gp38. Serially diluted phage suspensions were spotted onto LB plates containing 0.1% L-ara seeded with TY0807 cells harboring pBAD28 (–) or pBAD28-g38_T2_ (gp38_T2_). (**B**) Growth of DT2(∆g38) phages on *∆ompC_K12_* cells expressing gp38_PP01_ or chimeric gp38_T2-PP01_ in the presence of OmpC_O157_. Phage suspensions were spotted onto LB plates containing 0.1% L-ara seeded with *∆ompC_K12_* cells harboring pBAD33 (–) or pBAD33-OmpC_O157_ (+), along with pBAD24 (–), pBAD24-g38_PP01_ (gp38_PP01_), or pBAD24-g38_T2-PP01_ (gp38_T2-PP01_). (**C, D, E, and F**) Growth of DT2(∆g38) phages on *∆ompC_K12_* cells expressing various gp38_PP01_ loop mutants in the presence of OmpC_O157_. Phage suspensions were spotted onto LB plates containing L-ara and seeded with *∆ompC_K12_* cells harboring pBAD33-OmpC_O157_ plus plasmids expressing the indicated gp38 mutant constructs. “WT” denotes Gp38_T2-PP01_; “No” indicates no expression of gp38_T2-PP01_.

To determine which residues in gp38_PP01_ are responsible for interacting with OmpC_O157_, two mutant T4 phages—DT2 and DT2(Δg38)—were constructed. The DT2 phage genome contains genes *37* and *38* from the T2 phage in place of the corresponding T4 genes. In DT2(Δg38), gene *38* was deleted from the DT2 genome. DT2(∆g38) formed plaques on cells expressing T2 gp38 (gp38_T2_) from a plasmid but not on cells lacking the plasmid (Fig. 6A), confirming that gene *38* is essential for infection.

We next tested whether DT2(∆g38) could infect cells expressing gp38 from PP01 phage (gp38_PP01_) (Fig. 6B). For this, an *E. coli* K12 *ompC* deletion mutant (Δ*ompC_K12_*) expressing OmpC_O157_ from a plasmid was used as the indicator host. DT2(∆g38) failed to form plaques on cells expressing gp38_PP01_, likely due to incompatibility between T2 gp37 and PP01 gp38. In T2, the N-terminus of gp38 interacts with gp37 to form a functional tail fiber. To overcome this incompatibility, a plasmid was constructed encoding a chimeric gp38 protein (gp38_T2-PP01_), in which the N-terminal 113 amino acids of gp38_PP01_ were replaced with those of gp38_T2_. DT2(∆g38) successfully formed plaques on Δ*ompC_K12_* cells expressing this chimeric gp38 in an OmpC_O157_-dependent manner (Fig. 6B). This system thus enables direct functional analysis of gp38_PP01_ mutants via plaque-forming assays, providing a platform to assess their interaction with OmpC_O157_.

### Function of loop-E of GP38_PP01_ in adsorption

Cross-linking analysis indicated that Gly208 and Tyr230 in gp38_PP01_ interact with OmpC_O157_. To assess the functional relevance of these residues, we tested whether DT2(∆g38) could infect cells expressing gp38_T2-PP01_ variants with individual amino acid deletions (Fig. 6C). DT2(∆g38) formed plaques on cells expressing gp38_T2-PP01∆G208_ but not on those expressing gp38_T2-PP01∆Y230_, suggesting that Tyr230 plays a more critical role in adsorption. To further evaluate the role of Tyr230, we generated 19 substitution mutants at this position. Sixteen retained infectivity comparable to WT gp38_T2-PP01_ (Fig. 6D), indicating that most side chain substitutions at this position are tolerated. However, substitution with proline (Y230P) abolished infectivity, and glycine (Y230G) significantly reduced it, implying that the conformational integrity of loop-E is essential for function.

The Chai-1 predicted complex structure showed that loop-E of gp38_PP01_ penetrates the hydrophobic cleft formed by loops -5 and -7 of OmpC_O157_. Within this cleft, Ala228 of gp38_PP01_ appears to engage in hydrophobic interactions with Ile229 and Leu267 of OmpC_O157_ (Fig. S5A). Substitution of Ala228 with hydrophobic residues (Val or Leu) preserved infectivity (Fig. 6E), while replacement with a hydrophilic residue (Asp) or deletion of Ala228 abolished infectivity, confirming the importance of hydrophobic interactions at this site.

### Function of Tyr209 in loop-D of gp38_PP01_ for adsorption

Since PP01 can infect *E. coli* strains expressing OmpC_O157_ loop-5 mutants (P226A, ΔP226, L227G, and L227S) (Fig. 5F), loop-D of gp38_PP01_ may play a more significant role in host recognition. Although Gly208, located in loop-D, was identified as a cross-linked residue, its deletion or substitution with Ala did not impair infectivity (Fig. 6C), indicating that it is dispensable for adsorption. In contrast, mutations of the adjacent Tyr209 residue (Y209A, Y209S, and Y209L) completely abolished phage infectivity (Fig. 6F), suggesting that Tyr209 plays a critical structural role.

### Function of loop-B of GP38_PP01_ in adsorption

**Fig 7:**
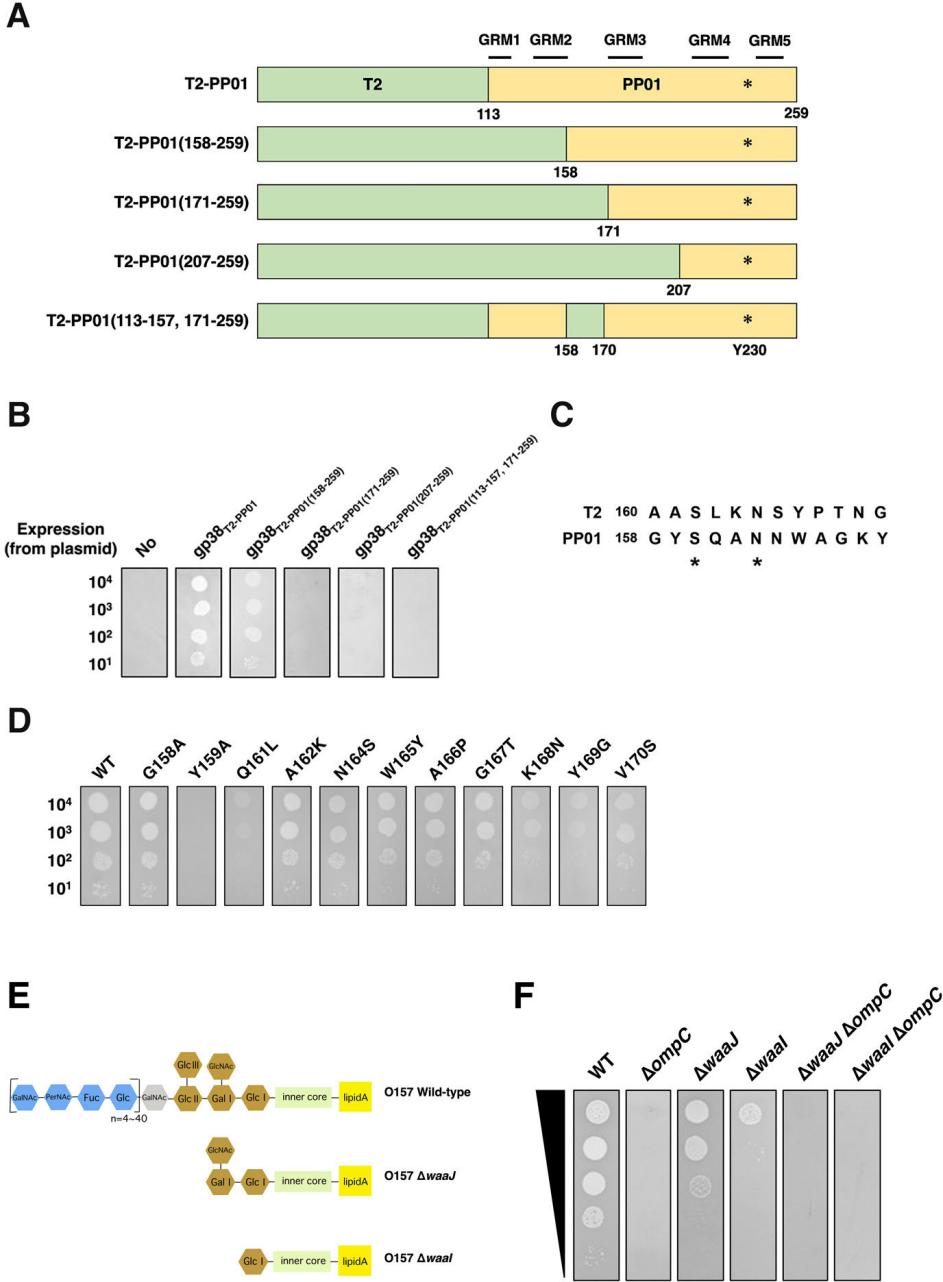
Role of gp38_PP01_ Loop-B in adsorption to OmpC_O157_. (**A**) Schematic of chimeric gp38 constructs. Green and yellow segments represent regions derived from T2 and PP01 gp38, respectively. Glycine-rich motifs are indicated above the sequences; asterisks denote the position of Tyr230. (**B**) Growth of DT2(∆g38) phage on ∆*ompC_K12_* cells expressing various chimeric gp38 constructs in the presence of OmpC_O157_. Serially diluted phage suspensions were spotted onto LB plates containing 0.1% L-ara seeded with *∆ompC_K12_* cells harboring pBAD33-OmpC_O157_ and plasmids expressing the indicated gp38 variants. “No” indicates no expression of gp38_T2-PP01_. (**C**) Amino acid sequence alignment of T2 gp38 (residues 160–172) and PP01 gp38 (residues 158–170). Asterisks indicate identical residues. (**D**) Growth of DT2(∆g38) phage on ∆*ompC_K12_* cells expressing gp38_PP01_ loop-B mutants in the presence of OmpC_O157_. “WT” denotes gp38_T2-PP01_. (**E**) Schematic representation of LPS structures in *E. coli* O157 WT and mutant strains. Horizontal lines represent backbone sugars; vertical lines indicate sugar branches. (**F**) Growth of PP01 phage on *E. coli* O157 WT and LPS-deficient mutant strains. Phage suspensions were spotted onto LB plates seeded with the indicated strains.

To identify additional gp38_PP01_ residues involved in interacting with OmpC_O157_, we constructed chimeric gp38 proteins in which C-terminal regions of gp38_PP01_ were replaced with corresponding sequences from gp38_T2_ (Fig. 7A). DT2(∆g38) phages formed plaques on cells expressing gp38_T2–PP01(158–259)_, but not on those expressing gp38_T2-PP01(171–259)_ or gp38_T2-PP01(207–259)_ (Fig. 7B), indicating that residues 158–170 of gp38_PP01_ are essential for adsorption. Supporting this, DT2(Δg38) failed to infect cells expressing gp38_T2-PP01(113–157, 171–259)_, in which residues 158–170 were replaced with the corresponding segment from gp38_T2_. This region forms loop-B at the distal tip of the long tail fiber.

To further assess the role of loop-B, each of the 11 residues spanning this region was individually replaced with the corresponding amino acid from gp38_T2_ (Fig. 7C). Among these, Y159A completely abolished infectivity, while Q161L, K168N, and Y169G reduced it (Fig. 7D). Residues Lys168 and Tyr169 project outward from the gp38_PP01_ crystal structure, and their substitutions had minimal effects on overall structure or OmpC binding. However, superposition of an LPS molecule from *Enterobacter cloacae* OmpE36 (PDB ID: 5FVN) (21), a homolog of OmpC_O157_, with the AlphaFold model of OmpC_O157_ revealed that Lys168 and Tyr169 lie near LPS sugar moieties (Fig. S5C). Consistently, *E. coli* O157 mutants with truncated LPS side chains (Δ*waaJ* and Δ*waaI*) exhibited similar infectivity patterns to those observed with the K168N and Y169G mutants (Fig. 7E and F), suggesting that Lys168 and Tyr169 contribute to LPS sugar recognition.

## DISCUSSION

The strict host specificity of bacteriophages is primarily determined by adsorption, which is mediated by interactions between RBPs, such as tail fibers, and bacterial surface receptors, including porins and LPS. Although the structures of various RBPs and receptor molecules have been elucidated, the molecular details of their interactions remain poorly understood. In this study, we investigated the molecular mechanism underlying the host specificity of phage PP01, which targets pathogenic *E. coli* O157:H7, focusing on the interaction between its tail fiber tip protein, gp38, and the host receptor OmpC.

We determined the crystal structure of the RBD of the gp38_PP01_ (gp38_PP01_RBD) (Fig. 1A). The overall structure consists of a poly-glycine type II (PG_II_) sandwich domain and protruding receptor recognition loops, closely resembling the gp38RBD of *Salmonella* phage S16 (15) (Fig. 1B). Guided by this structural information, UV cross-linking analysis using pBPA identified Gly208 in loop-D and Tyr230 in loop-E of gp38_PP01_ as cross-linking sites with OmpC_O157_ (Fig. 2A). LC-MS/MS analysis further revealed that Gly208 cross-linked with Ser225 and Pro226 in loop-5 of OmpC_O157_, while Tyr230 interacted with residues Val304–Arg308 in loop-7 of OmpC_O157_ (Tables 2 and 3). The GVIN motif (Gly303-Val304-Ile305-Asn306), present in loop-7 of OmpC_O157_ but absent in OmpC_K12_ (which is not susceptible to PP01), was identified as a key determinant for gp38 recognition. Using cross-linking data as spatial restraints, we generated a gp38_PP01_RBD–OmpC_O157_ complex model via distance-constrained structural prediction. Notably, models generated without these constraints erroneously predicted gp38 binding to the other sites of OmpC (Fig. 3 and Fig. S3C). In contrast, the distance-restraint model showed gp38_PP01_ receptor recognition loops interacting with the shape and hydrophobic surface of extracellular OmpC_O157_ (Fig. 3B; Movie S1). Several experimental findings support this model. First, the GVIN motif in loop-7 was shown to be essential for PP01 binding and infection (Fig. 4C and D). Second, deletion of individual residues within the GVIN sequence revealed that Asn306 and Gly307 are critical for PP01 adsorption (Fig. 5B). AlphaFold-predicted structures of the OmpC_O157_ mutants present altered loop-7 main chain conformations and deformation of the cleft between loop-7 and -5, likely impeding the insertion of gp38_PP01_ loop-E (Fig. S6). Notably, mutations in loop-7 and loop-5 of *Shigella* OmpC also impair the adsorption of phages Sf22 and KRT47 (14), and the loop-5 composition of *Salmonella* OmpC is similarly critical for phage S16 infection (15). These observations support a conserved structural principle underlying phage–OmpC interactions across diverse bacterial species.

Mutational analysis of gp38 is inherently challenging due to the difficulty of introducing targeted mutations into the phage genome while retaining viability. To overcome this limitation, we established a functional assay system using the chimeric phage DT2(∆38), which lacks gene *38* and can be complemented in *trans* (Fig. 6A and B). Using this system, we demonstrated that the deletion of Tyr230 or its substitution with proline, both located in loop-E of gp38_PP01_, completely abolished infectivity (Fig. 6C). In contrast, most other amino acid substitutions at Tyr230 retained infectivity (Fig. 6D), indicating that the conformational integrity of loop-E, rather than the side chain of Tyr230 itself, is critical for adsorption. Further analysis of Ala228, located at the tip of loop-E, revealed its role in mediating hydrophobic interactions within the cleft formed by loops -5 and -7 of OmpC_O157_, consistent with its structural placement in the predicted complex (Fig. S5A).

Similarly, mutations at Tyr209 in loop-D led to complete loss of infectivity (Fig. 6F). Structural predictions suggest that loss of the Tyr209 side chain alters loop-E positioning, thereby impairing its ability to interact with the OmpC-binding cleft (Fig. S5A). Notably, the Y159A mutation in loop-B also abolished infectivity (Fig. 7D), despite the absence of direct cross-linked interactions at this site. Structural modeling indicates that Y159A induces conformational changes that affect Tyr209 and, consequently, loop-E orientation (Fig. S5A). Although the membrane abundance of OmpC was reduced in LPS mutants (Fig. S4C), combined evidence from LPS superposition on OmpC, mutational analysis of gp38_PP01_ residues Lys168 and Tyr169, and *E. coli* O157 LPS mutants (Δ*waaJ* and Δ*waaI*) suggests that loop-B residues may also contribute to recognition of the LPS sugar moiety (Fig. 7D through F and Fig. S5C) (22).

Collectively, these findings demonstrate that PP01 host specificity is primarily determined by the interaction of loop-E of gp38_PP01_ with the cleft formed by loops -7 and -5 of OmpC_O157_. In particular, the four-residue insertion comprising the GVIN motif in loop-7 is a key structural determinant of this specificity. Additionally, loops -B and -D of gp38 appear to modulate loop-E conformation and may influence host recognition indirectly. These results strongly support the validity of the gp38–OmpC complex model, which was generated using molecular surface geometry and hydrophobicity as guiding parameters. Notably, this modeling strategy also successfully produced a plausible interaction model for *Salmonella* phage 16 gp38_S16_RBD and OmpC, consistent with previous adsorption assays (Fig. S7 and Movie S2) (15).

The molecular insights gained in this study advance our understanding of phage–host interactions and establish a framework for engineering phages with altered host specificity. Such strategies hold considerable potential for developing targeted antimicrobial therapies against antibiotic-resistant bacteria. Future research should focus on directly resolving the structures of T2-type phage RBP–host receptor complexes using high-resolution techniques such as cryo-electron microscopy, as exemplified by the T5 phage RBP pb5 in complex with *E. coli* FhuA (23). Additionally, further exploration of the dynamic nature of these interactions will provide a more comprehensive understanding of host recognition mechanisms.

## MATERIALS AND METHODS

### Crystallization of sfGFP-PP01gp38C-his

Purified sfGFP-PP01gp38C-his was concentrated to approximately 20 mg/mL using an Amicon Ultra-4 centrifugal filter device (30 kDa cutoff, Merck, Germany). Diffraction quality crystals were obtained by hanging-drop vapor diffusion method at 20°C with a reservoir solution containing 10% (wt/vol) PEG 8000, 0.1 M Na-HEPES-MOPS (pH 7.5), 3 mM CaCl_2_, 3 mM MgCl_2_, 20% (vol/vol) ethylene glycol. X-ray diffraction data sets were collected from flash-frozen (under a liquid nitrogen stream) crystals at 100 K on the Photon Factory (PF) beamline AR-NE3A using a PILATUS 2M-F single-photon counting pixel detector. Data were collected up to 2.1 Å resolution (Table 1).

### Structure determination of sfGFP-PP01gp38C-his

Diffraction data were indexed and integrated by XDS (24) and scaled using SCALA (25) (Table 1). Phases were determined by molecular replacement using Phaser (26), with the atomic coordinates of sfGFP (PDB ID: 4J8A) and the RBD of phage S16 gp38 (PDB ID: 6F45) as search models. Model building was performed with Coot (27), and refinement was carried out using Phenix (28) and Coot (Table 1).

### UV cross-linking of pBPA-labeled PP01-gp38C and OmpC_O157_

*E. coli* BL21 (DE3) cells were co-transformed with (i) 6×His-PP01gp38C-pETDuet vectors harboring 19 unique amber codon mutations and (ii) pEVOL-pBpF, which encodes an orthogonal tRNA/tRNA synthetase pair for *in vivo* incorporation of pBPA (29). Cells were cultured in Luria-Bertani (LB) medium containing 0.4% glucose at 30°C until OD_600_ reached 0.5, harvested, and resuspended in LB medium supplemented with 0.02% arabinose and 0.1% pBPA. After 2-h incubation at 30°C, protein expression was induced with 1 mM isopropyl β-D-1-thiogalactopyranoside for an additional 2 h at 30°C. Cells were harvested by centrifugation and stored at −20°C. Frozen cell pellets were resuspended in phosphate-buffered saline (PBS) and disrupted by micro-tip sonication. Lysates were centrifuged at 20,000 × *g* for 30 min, and the supernatant was mixed with the membrane fraction of *E. coli* O157:H7. This mixture was irradiated with 365 nm UV light for 20 min at 4°C using a B-100AP UV lamp (UVP, Analytik Jena, Germany), followed by centrifugation at 20,000 × *g* for 60 min. The resultant pellet was resuspended in PBS and solubilized with 0.5 M NaCl and 1% SDS. His-tagged and cross-linked proteins were purified using Ni-NTA agarose spin columns (Qiagen, Germany). After three washes with PBS containing NaCl and SDS, bound proteins were eluted with 500 mM imidazole in the same buffer.

### Protein digestion and peptide preparation for LC-MS/MS analysis

Eluted protein samples were reduced with 25  mM dithiothreitol for 15 min and alkylated with 50 mM iodoacetamide for 15 min at 25°C to block cysteine residues. Samples were mixed with 5× SDS PAGE loading buffer and separated on 8% wide-range polyacrylamide gels. Gels were fixed in 40% methanol and 10% acetic acid, then stained with Bio-Safe Coomassie stain (Bio-Rad, USA). Stained bands were excised using a clean razor blade, sliced into small pieces, and transferred to 1.5 mL Protein LoBind Tubes (Eppendorf, Germany). Gel pieces were washed at least twice with destaining solution (50 mM ammonium bicarbonate, 30% acetonitrile) until transparent, followed by three rounds of dehydration using a solution of 50 mM ammonium bicarbonate and 60% acetonitrile. Gel pieces were then dried using a centrifugal evaporator. For trypsin digestion, dried gel pieces were rehydrated in 75 μL of 50 mM ammonium bicarbonate containing 0.01 mg/mL Trypsin Gold (Promega, USA) and incubated overnight at 37°C. Digested peptides were extracted sequentially using 75 µL of 50% acetonitrile, 75 μL of 0.1% trifluoroacetic acid (TFA), and 75 μL of 100% acetonitrile. Each extraction step involved 15 min of shaking at 25°C followed by supernatant collection. All extracts were pooled and dried using a centrifugal evaporator. For the G208B sample, peptides were reconstituted in 25 μL of 2 M urea and 25 μL of 50 mM ammonium bicarbonate containing 0.03 mg/mL Glu-C (Sequencing Grade, Promega), followed by 8-h incubation at 37°C. For the Y230B sample, peptides were dissolved in 47 μL of 32 mM HCl, then mixed with 2.5 μL of 0.2 mg/mL ProAlanase (Mass Spec Grade, Promega), and incubated overnight at 37°C. After the second protease digestion, samples were acidified with 2 μL of 10% TFA and supplemented with 1 μL of acetonitrile. Peptides were desalted using C18 StageTips (Nikkyo Technos, Japan), eluted with 0.1% TFA in 80% acetonitrile, dried using a centrifugal evaporator, and redissolved in 0.1% TFA with 2% acetonitrile for LC-MS/MS analysis.

### LC-MS/MS analysis of cross-linked products

LC-MS/MS analysis was performed using a Q-Exactive hybrid quadrupole–Orbitrap mass spectrometer coupled with an Easy-nLC 1000 nano-HPLC system (Thermo Fisher Scientific, USA). For peptide separation, a trap column (2 cm × 75 µm) packed with 3 µm C18 silica particles (Thermo Fisher Scientific) was used in conjunction with an analytical column (12.5 cm × 75 µm) packed with 3 µm C18 silica particles (Nikkyo Technos, Japan). The flow rate was maintained at 300 nL/min. Peptides were separated using a linear gradient of 10%–50% acetonitrile over 40 min in the presence of 0.1% formic acid. MS data were acquired in data-dependent acquisition (DDA) mode using Xcalibur 4.0 (Thermo Fisher Scientific). The DDA parameters were as follows: resolution of 70,000 for full MS scans and 17,500 for MS/MS scans; automatic gain control target of 3.0 × 10⁶ for MS and 5.0 × 10⁵ for MS/MS; maximum injection time (IT) of 60 ms for both scans; MS scan range of 310–1,500 m/z. The top 10 precursor ions from each full MS scan were selected for fragmentation using an isolation window of 3.0 *m/z* and a normalized collision energy (NCE) of 30. Only precursor ions with charge states of +2 to +6 were selected, and dynamic exclusion was set to 30 s. Cross-linked peptides were identified using pLink version 2.3.9 (30). The pBPA residue was defined as an unnatural amino acid with the formula C_16_H_15_NO_3_–H_2_O (251.0946 Da), specifically incorporated at position 208 or 230 of the Gp38 sequence. Cross-links were defined between pBPA and any of the 20 standard amino acids, with a linker mass of 0.000 Da.

### Construction of DT2 and DT2∆g38 mutant phages

DT2 is an engineered T4 phage in which genes *37* and *38* have been replaced with those from T2 phage. To construct DT2, T2 genomic DNA was digested with *Nde*I (Toyobo, Japan), and ~10 kb DNA fragments were purified. *E. coli* B strain B40su1 was grown in LB medium at 37°C to a density of 3 × 10⁸ cells/mL. T4 phage mutant harboring amber mutations in genes *37* and *38* was added at a multiplicity of infection (MOI) of 5. After 5 min of adsorption, infected cells were harvested and washed several times with ice-cold 10% glycerol. The purified T2 DNA fragments were electroporated into these cells, which were then plated on LB agar seeded with *E. coli* B strain BB. Eight plaques were observed. Phages isolated from these plaques were replated on LB agar seeded with ∆*ompC* cells, and four phages capable of proliferation were isolated. Sequence analysis of the genomic regions surrounding genes *37* and *38* confirmed the replacement of the T4 gene segments with those from T2 phage in all isolates, verifying the construction of the DT2 phage. To generate DT2∆g38, a DNA fragment linking gene *37* to gene *T* was constructed by PCR. First, a fragment containing a portion of gene *37* was amplified from T2 genomic DNA using primers T2g37-1up and T2g37-2dw. A second fragment containing a portion of gene *T* was amplified using primers T2t-2up and T2t-1dw. These two fragments were assembled into a single contiguous fragment by overlap-extension PCR. *E. coli* K-12 strain TY0807 was grown in LB medium at 37°C to a density of 3 × 10⁸ cells/mL. A DT2 phage mutant carrying the mutations *nd28*(*denA^-^*), *rIIPT8* (*denB*^-^), and *amSE1243*(T2g37^-^) was added at an MOI of 5. After 5 min of adsorption, cells were harvested and washed with ice-cold 10% glycerol. The assembled PCR fragment was electroporated into these competent cells, which were then plated on LB agar seeded with TY0807. The resulting DT2∆g38 *nd28*(*denA^-^*) *rIIPT8* (*denB*^-^) phage was selected on plates seeded with TY0807 cells harboring either pBAD28-g38_T2_ or the empty vector pBAD28. The phage formed plaques only on cells expressing g38_T2_ (pBAD28-g38_T2_) but not on cells containing the empty vector, confirming the successful deletion of gene *38* and its requirement for complementation. Finally, the *nd28*(*denA^-^*) and *rIIPT8* (*denB*^-^) mutations were removed by backcrossing with DT2 phage, and the resulting DT2∆g38 was isolated.

### Bacteriophage adsorption assay

*E. coli* cells were grown in LB medium at 37°C to a density of 3 × 10⁸ cells/mL, with or without supplementation of 0.1% L-arabinose (L-ara). T4 phage was added at an MOI of 0.01. At designated time points, aliquots were withdrawn and immediately diluted in BS buffer (8% NaCl, 10 mM potassium phosphate buffer, pH 7.4) saturated with chloroform to inactivate residual bacterial cells. The number of unadsorbed phages was quantified by plating serial dilutions on agar plates seeded with an appropriate *E. coli* indicator strain. The percentage of unadsorbed phages was calculated by setting the initial phage input as 100%. Adsorption assays were performed in triplicate, and results are presented as mean ± standard deviation (SD).

## Data Availability

The atomic models for the sfGFP-gp38PP01RBD fusion protein have been deposited in the PDB with accession code 9V85.

## SUPPLEMENTAL MATERIAL

Supplemental material mbio.02110-25-s0001.pdf Figures S1-S7, Tables S1-S4, supplemental materials and methods, and legends for Movies S1 and S2.

Movie S1 mbio.02110-25-s0002.mp4 Predicted gp38_PP01_RBD-OmpC_O157_ complex.

Movie S2 mbio.02110-25-s0003.mp4 Proposed gp38_S16_RBD-OmpC*_S. typhi_* complex model.

ASM does not own the copyrights to Supplemental Material that may be linked to, or accessed through, an article. The authors have granted ASM a non-exclusive, world-wide license to publish the Supplemental Material files. Please contact the corresponding author directly for reuse.
