# Supplementary material for: Structural and functional insights into the interaction between a PP01 phage gp38 tail fiber tip and an Escherichia coli OmpC receptor
Source: mBio. 2026 Jan 9;17(2):e02110-25. doi: 10.1128/mbio.02110-25 (PMC12892980; doi:10.1128/mbio.02110-25)
Supplement: Supplemental material — Figures S1-S7, Tables S1-S4, supplemental materials and methods, and legends for Movies S1 and S2. [file mbio.02110-25-s0001.pdf]

SUPPLEMENTAL MATERIALS

Structural and functional insights into the interaction between an PP01 phage gp38 tail fiber tip and an *Escherichia coli* OmpC receptor

Haruka Terasaki[a], Aleksandar Zdravković[b,c], Tatsuya Niwa[b,c], Ayaka Washizaki[d], Marina Kawaguchi[e], Tetsuro Yonesaki[e], Shuji Kanamaru[b]#, Yuichi Otsuka[a]#

[a]Department of Biochemistry and Molecular Biology, Graduate School of Science and Engineering, Saitama University, Saitama, Japan

[b]Department of Life Science and Technology, Institute of Science Tokyo, Kanagawa, Japan

[c]Institute of Integrated Research, Institute of Science Tokyo, Kanagawa, Japan

[d]Laboratory of Phage Biologics, Graduate School of Medicine, Gifu University, Gifu, Japan

[e]Department of Biological Sciences, Graduate School of Science, Osaka University, Osaka, Japan

#Corresponding author:

Shuji Kanamaru, skanamar@bio.titech.ac.jp

Yuichi Otsuka, otsukay@mail.saitama-u.ac.jp

SUPPLEMENTAL FIGURES

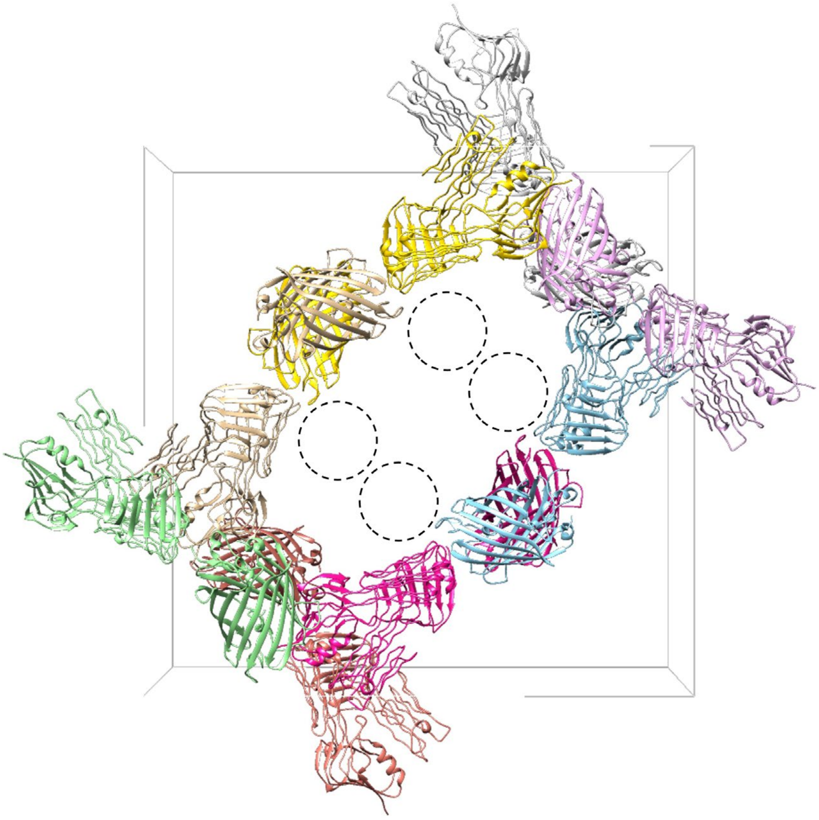

Fig. S1 Molecular packing of sfGFP-gp38$_{PP01}$RBD in the crystal lattice

Dotted circles indicate regions of missing electron density corresponding to one of the sfGFP (superfolder green fluorescent protein) fusion molecules.

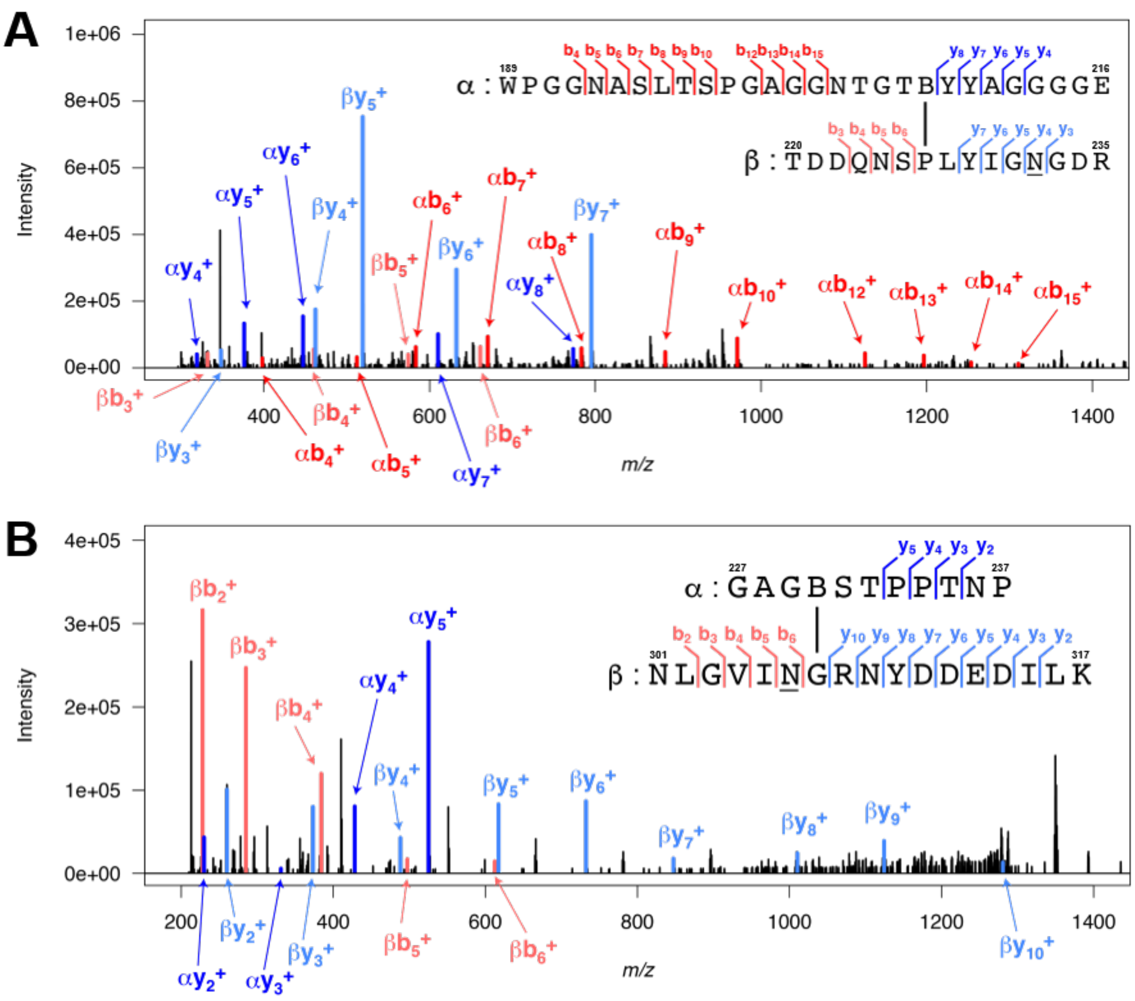

Fig. S2 Representative MS/MS spectra of cross-linked peptides

MS/MS spectra of cross-linked peptides between loop-D of gp38-G208B and loop-5 of OmpC (A), and loop-E of gp38-Y230B and loop-7 of OmpC (B). The pBPA residue is indicated by the letter "B". The numbers on top of the peptide sequences indicate the residue numbers of each proteins. Underlined residues denote deamidated asparagine. Signal intensities were obtained from QualBrowser (Thermo Fisher Scientific), and fragment ions were manually re-annotated based on pLink software output.

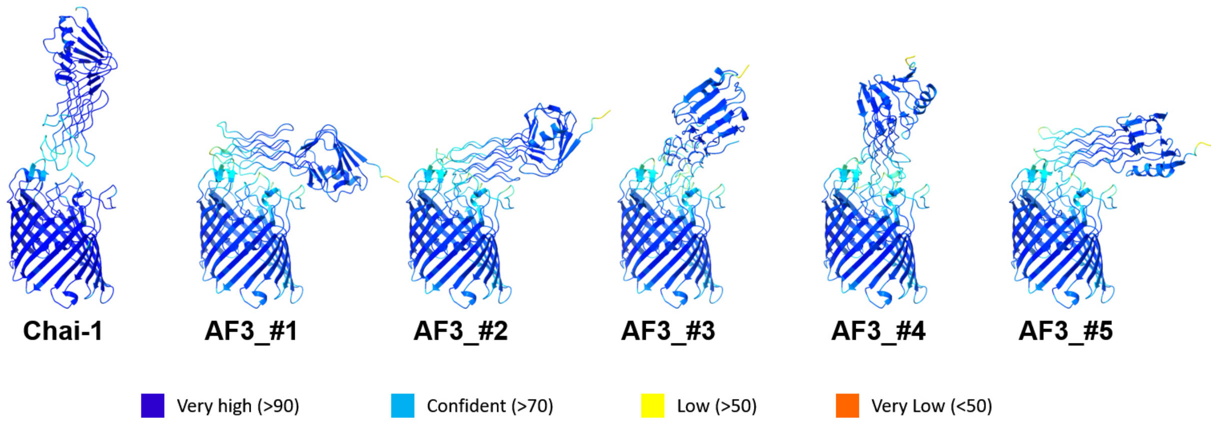

Fig. S3 Confidence of predicted gp38C$_{PP01}$RBD-OmpC$_{O157}$ structures

The Chai-1 (distance constrained) and the top five AlphaFold3 predicted models of gp38$_{PP01}$RBD-OmpC$_{O157}$ colored by pLDDT values, which measures the confidence of atomic positions relative to its neighboring atoms.

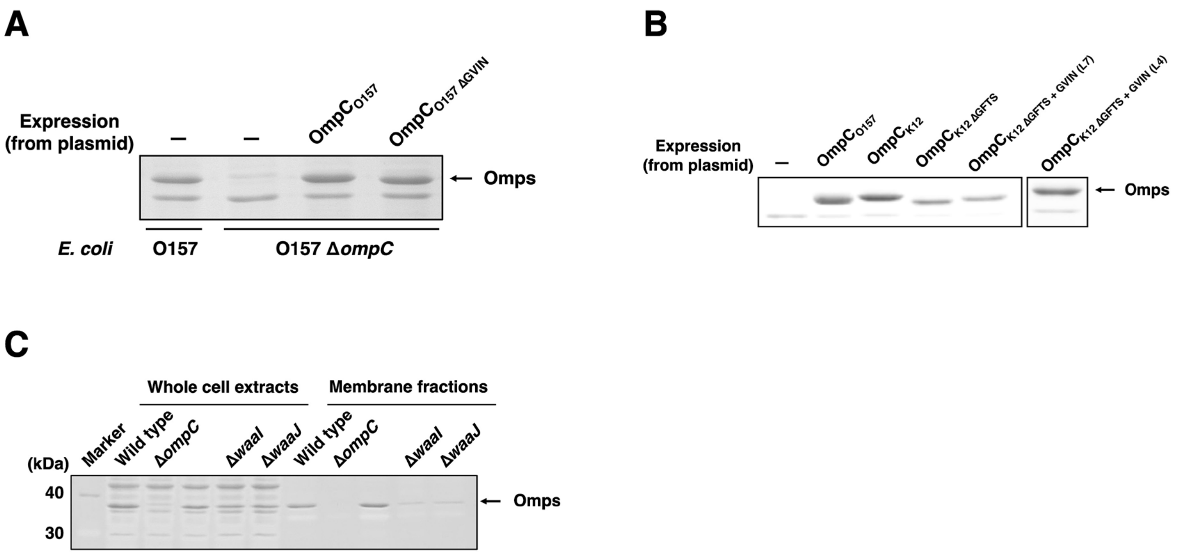

Fig. S4 Expression of various OmpC variants in the membrane fraction

(A) SDS-PAGE analysis of membrane proteins extracted from *E. coli* O157 wild-type or Δ*omp*C_{O157} cells harboring pBAD33-$OmpC_{O157}$ or pBAD33-$OmpC_{O157(\Delta GVIN)}$. "–" indicates cells lacking plasmid. "Omps" refers to outer membrane proteins C and F. (B) SDS-PAGE analysis of membrane proteins from Δ*ompC*$_{O157}$ cells expressing $OmpC_{O157}$, $OmpC_{K12}$, $OmpC_{K12(\Delta GFTS)}$, $OmpC_{K12(\Delta GFTS+GVIN(L7))}$, or $OmpC_{K12(\Delta GFTS+GVIN(L4))}$. (C) SDS-PAGE analysis of whole-cell lysates and membrane fractions from *E. coli* O157 wild-type and the indicated lipopolysaccharide (LPS) mutant strains.

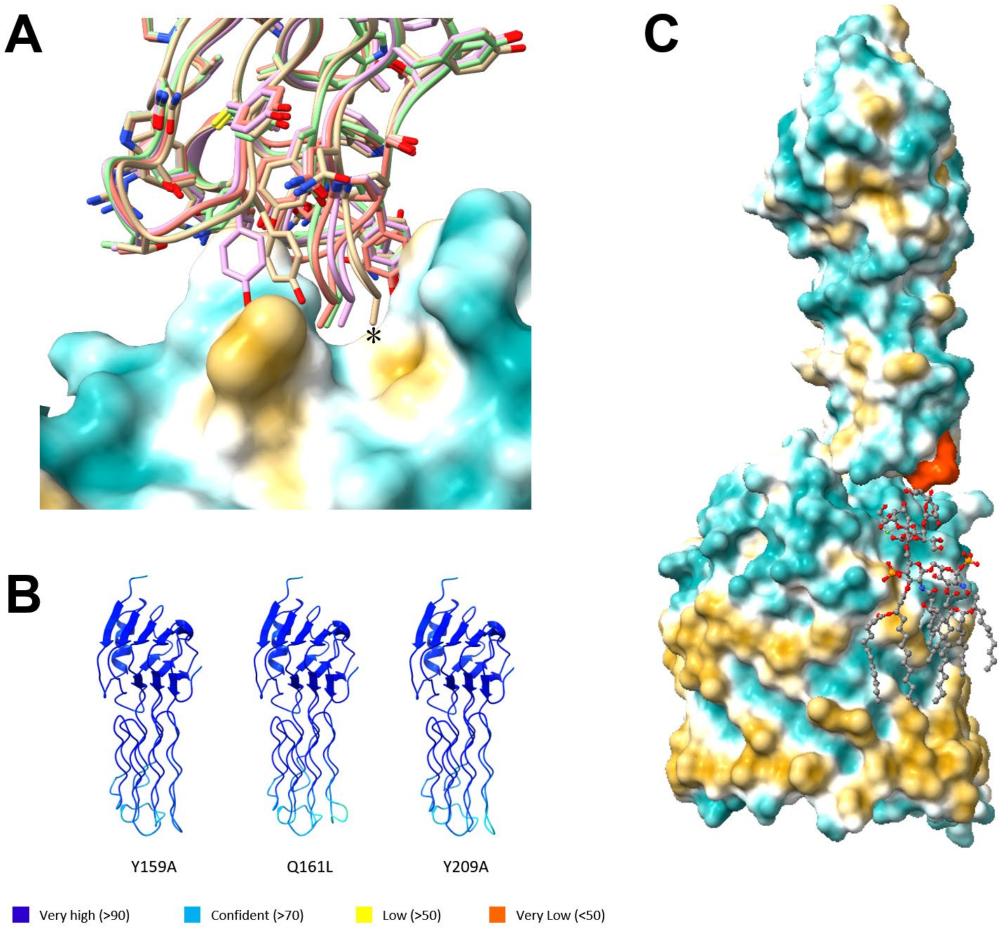

Fig. S5 Predicted gp38C$_{PP01RBD}$ mutants' structures on OmpC$_{O157}$ structure

(A) Close-up view of predicted gp38$_{PP01RBD}$ mutant structures superimposed onto the proposed complex model. OmpC$_{O157}$ is shown as a hydrophobic surface. Wild-type, Q161L, Y159A, and Y209A mutants are represented in tan, light purple, emerald green, and pink, respectively. As asterisk marks residue Ala228 of wild-type gp38$_{PP01}$.Structures of gp38C$_{PP01RBD}$ mutants were predicted using AlphaFold3. (B) The wild-type and the three predicted models of OmpC$_{O157}$ colored by pLDDT values, which measures the confidence of atomic positions relative to its neighboring atoms. (C) Superimposition of a lipopolysaccharide (LPS) model (gray ball-and-stick) from *Enterobacter cloacae* OmpE36 (PDB ID: 5FVN) onto the proposed gp38–OmpC$_{O157}$ complex. Loop-B residues Lys168 and Tyr169 of gp38$_{PP01}$ are highlighted in orange.

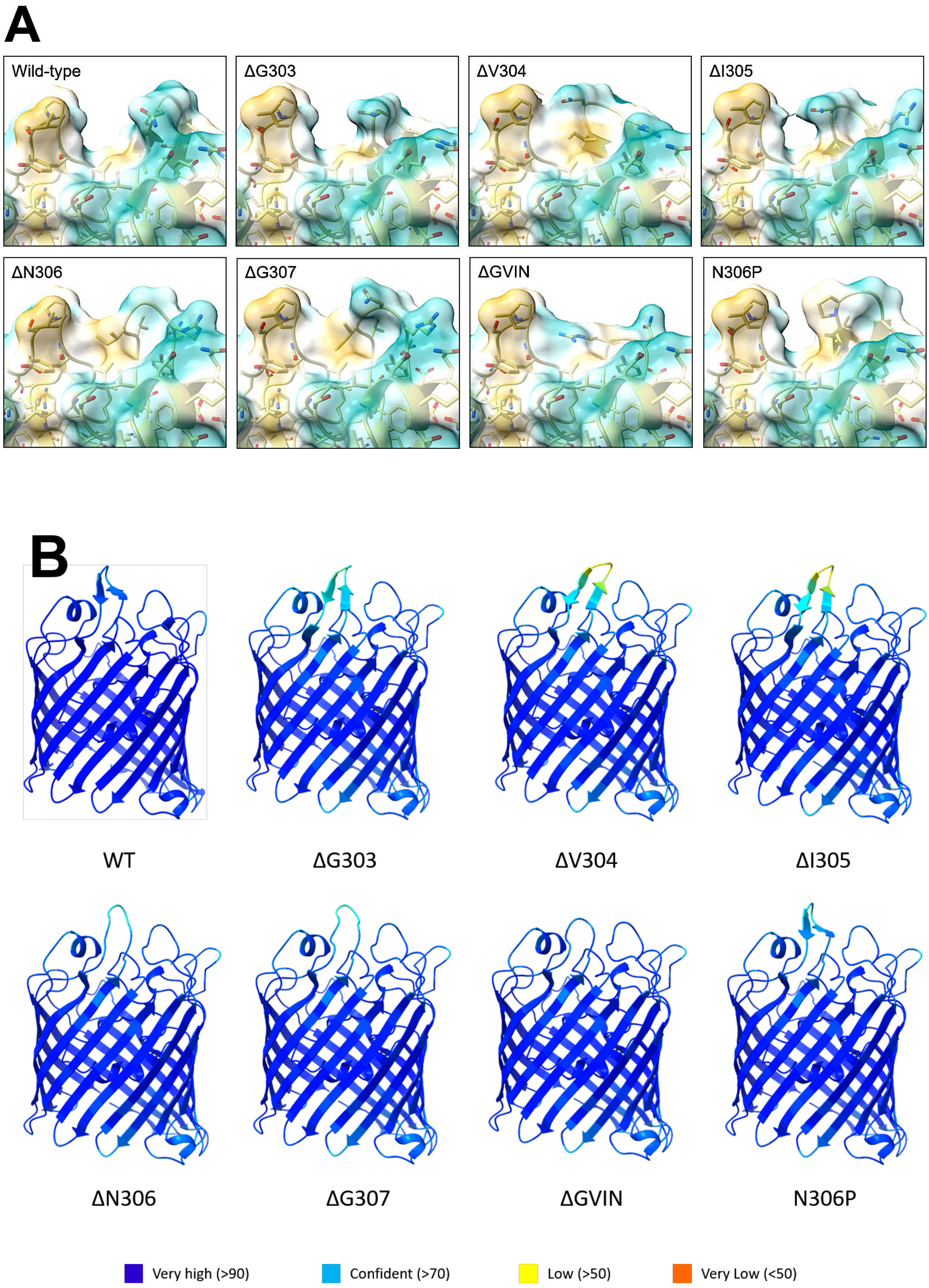
A
Wild-type
ΔG303
ΔV304
ΔI305
ΔN306
ΔG307
ΔGVIN
N306P
B
WT
ΔG303
ΔV304
ΔI305
ΔN306
ΔG307
ΔGVIN
N306P
Very high (>90)
Confident (>70)
Low (>50)
Very Low (<50)

Fig. S6 Predicted OmpC$_{O157}$ loop-7 mutants' structures

(A) Close-up views of the cleft formed between loop-5 and loop-7 of OmpC$_{O157}$, visualized as a semi-transparent hydrophobicity surface with overlaid stick models. Structures of OmpC mutants were predicted using AlphaFold3. (B) The wild-type and seven predicted models of OmpC$_{O157}$ colored by pLDDT values, which measures the confidence of atomic positions relative to its neighboring atoms.

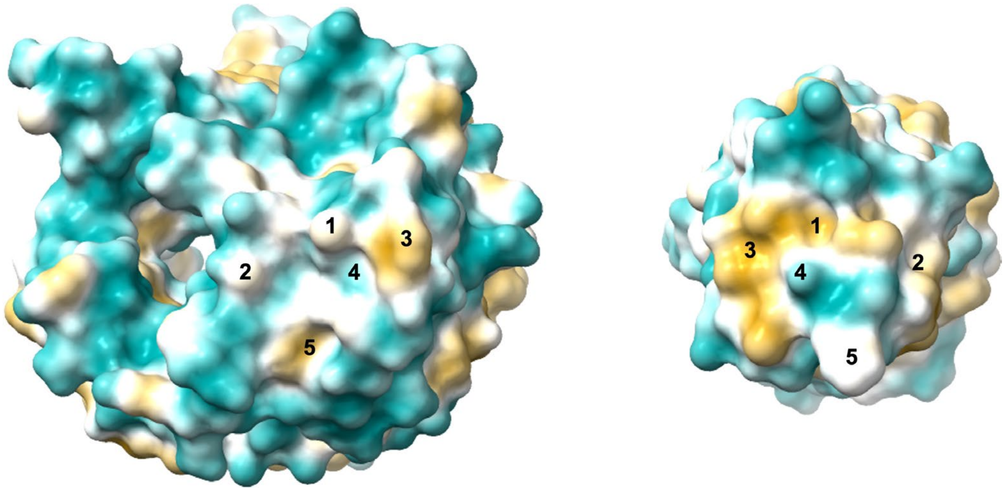

Fig. S7 Proposed interaction of the gp38$_{S16}$RBD-OmpC$_{\textit{S. typhi}}$ complex

Hydrophobic surface representation showing a bottom view of the phage S16 gp38RBD (right) and a top view of the *Salmonella typhimurium* OmpC monomer (left). Hydrophobicity is rendered from cyan (hydrophilic) to yellow (hydrophobic). Numbered labels indicate corresponding "hill" and "valley" regions on each protein surface predicted to mediate interaction.

SUPPLEMENTAL MOVIES

Movie S1 Predicted gp38$_{PP01}$RBD-OmpC$_{O157}$ complex

The OmpC$_{O157}$ monomer is shown in silver with its hydrophobic surface rendered from cyan (hydrophilic) to yellow (hydrophobic). gp38$_{PP01}$RBD is shown in tan.

Movie S2 Proposed gp38$_{S16}$RBD-OmpC$_{\textit{S. typhi}}$ complex model

The OmpC$_{\textit{S. typhi}}$ monomer is shown in silver, with residues 227-NAR-229 highlighted in yellow. The hydrophobic surface is rendered from cyan (hydrophilic) to yellow (hydrophobic). gp38$_{S16}$RBD is shown in tan.

SUPPLEMENTAL MATERIALS AND METHODS

Escherichia coli strains and phages

The *E. coli* strains used in this study are listed in Table S1. Wild-type phages include T4D (1), T2L (2), and PP01 (3).

Construction of plasmids

The primers used in this study are listed in Table S2. All restriction enzymes were purchased from Toyobo (Osaka, Japan) and Takara Bio (Shiga, Japan). To construct pBAD28-$g38_{T2}$ or pBAD24-$g38_{PP01}$, DNA fragments encoding gene *38* were amplified by PCR using genomic DNA from T2 or PP01 phage as templates and primers T2g38-1up/T2g38-dw or YO-949/YO-950, respectively. PCR products were digested with *Sal*I and *Hin*dIII (for $g38_{T2}$) or *Kpn*I and *Pst*I (for $g38_{PP01}$), and ligated into the corresponding sites of pBAD28 or pBAD24. pBAD33-$OmpC_{K12\Delta GFTS}$ was constructed as follows: one fragment encoding the N-terminal region (residues 1–180) of *E. coli* K-12 OmpC was amplified from pBAD33-$OmpC_{K12}$ (4) using primers YO-1008 and YO-1011. Another fragment encoding the C-terminal region (residues 185–367) was amplified using primers YO-1009 and YO-1010. The two fragments were joined by overlap-extension PCR using primers YO-1008 and YO-1009, generating a GFTS-deleted OmpC variant. The resulting product was digested with *Kpn*I and *Hin*dIII and ligated into pBAD33. pBAD24-$g38_{T2\text{-}PP01}$, a chimeric construct, was assembled using Gibson Assembly. The N-terminal region (residues 1–113) of the T2 gp38 was amplified from pBAD28-$g38_{T2}$ using primers YO-990 and YO-991. A second fragment from pBAD24-$g38_{PP01}$ was amplified using primers YO-988 and YO-989. Both PCR products were assembled using Gibson Assembly Master Mix (New England BioLabs, USA). Plasmids encoding wild-type or mutant $OmpC_{K12}$, $OmpC_{O157}$, or gp38 variants were constructed using a KOD-Plus-Mutagenesis Kit (Toyobo, Japan) or Gibson Assembly. Primers and templates used for site-directed

mutagenesis are listed in Tables S3 and S4. All mutations were confirmed by Sanger sequencing using primers YO-82 or YO-155 (5). To express the receptor binding domain (RBD; residues 41–259) of PP01 gp38, the corresponding DNA fragment was amplified using primers pp01-g38G41-EcoRI(+) and pp01-nonSTP-Hind3(−) (Table S2), digested with *Eco*RI and *Hin*dIII, and cloned into the pET22-sfGFP (a gift from T. Takahashi). The resulting plasmid, sfGFP-PP01_gp38C-his-pET22, encodes the RBD fused to an N-terminal sfGFP and a C-terminal His-tag. To generate His-PP01_gp38C-pETDuet, the same DNA fragment was amplified using primers pp01-g38G41-EcoRI(+) and pp01-STP-Hind3(−), digested and cloned into the pETDuet-1 vector (Merck, Germany). Site-specific amber codons for pBPA incorporation were introduced by inverse PCR using the KOD-Plus-Mutagenesis Kit (Toyobo, Japan). Primers and template DNAs used for mutagenesis are listed in Tables S2 and S3. pEVOL-pBpF, used for pBPA incorporation, was a gift from Peter Schultz (Addgene plasmid # 31190 ; http://n2t.net/addgene:31190 ; RRID:Addgene_31190) (6).

Expression and purification of sfGFP-gp38C-his

The fusion protein sfGFP–PP01gp38C-his was expressed in *E. coli* BL21(DE3) by induction with isopropyl β-D-1-thiogalactopyranoside (IPTG). Cells from 1 L of LB medium were harvested and resuspended in 30 mL of buffer A (100 mM Tris-HCl, pH 8.0; 25 mM imidazole) on ice. Cell lysis was performed by sonication (Branson Sonifier 250), and the lysate was clarified by centrifugation at 20,000 × *g* for 30 min. The supernatant was applied to a 5 mL HisTrap HP Ni-affinity column (GE Healthcare, USA) pre-equilibrated with buffer A. Elution was carried out using a linear gradient of imidazole from 25 mM to 500 mM. To prevent protein aggregation, EDTA was added to each eluted fraction to a final concentration of 5 mM. Pooled fractions were applied to a 5 mL HiTrap Q HP anion exchange column (GE Healthcare) pre-equilibrated with buffer B (20 mM Tris-HCl, pH 8.0). Elution was performed using a linear

NaCl gradient of 0 to 1 M. Fractions containing sfGFP–PP01gp38C-his were further purified by size-exclusion chromatography using a HiLoad 16/600 Superdex 200 pg column (GE Healthcare), equilibrated with buffer C (20 mM Tris-HCl, pH 8.0; 100 mM NaCl).

Preparation of membrane fraction from *E. coli* O157:H7 for cross-link analysis

E. coli O157:H7 was grown overnight in LB medium. Cells were harvested by centrifugation at 5,000 × *g* for10 min and resuspended in 20 mM Tris-HCl (pH 8.0) containing 50 mM NaCl. Cells were disrupted by sonication, and debris was removed by centrifugation at 5,000 × *g* for 10 min. The supernatant was centrifuged at 20,000 × *g* for 30 min to obtain the membrane fraction as a pellet.

Protein structure prediction

Monomeric protein structures were predicted using AlphaFold 3 via AlphaFold server (7). The distance-restrained $Gp38_{PP01}$RBP–$OmpC_{O157}$ complex model was generated using Chai-1, accessed through its web interface (8).

Sequence Alignments and Molecular Graphics

Figures were rendered using UCSF ChimeraX (9). In Figure 1C, receptor-binding domain sequences of phage gp38 were aligned using ClustalOmega (10) and visualized using SnapGene Viewer (Insightful Science; available at snapgene.com).

Detection of OmpC in the membrane fraction

E. coli cells were grown in 5 mL LB medium with or without 30 μg/mL chloramphenicol and 0.2% L-arabinose until the OD_{600} reached approximately 0.5. Cells were harvested, washed once with PBS, and resuspended in 1 mL of 10 mM Tris-HCl (pH 7.5). Cell lysis was performed by sonication or lysozyme treatment, and debris was removed by centrifugation at

2,300 × *g* for 5 min. The supernatant was then centrifuged at 20,000 × *g* for 1 h, and the resulting membrane pellet was resuspended in 40 μL of 10 mM Tris-HCl (pH 7.5) and stored. Proteins were separated by SDS-PAGE by 12.5% gels and visualized using Coomassie Brilliant Blue (CBB) staining.

SUPPLEMENTAL TABLES

TABLE S1 The *Escherichia coli* strains used in this study

Strains	Genotype	Source/Reference
TY0807	*sup^0 araD139 hsdR ΔlacX74 rpsL $araD^+$*	Koga *et al*. 2011 (11)
TY0807 Δ*ompC*	TY0807 Δ*ompC*::*kan*	Suga *et al*. 2021 (5)
O157:H7	Wild-type (ATCC43888)	Morita *et al*. 2002 (3)
TY0731	O157:H7 Δ*waaI*::*cat*	Washizaki *et al*. 2016 (4)
TY0732	O157:H7 Δ*waaJ*::*cat*	Washizaki *et al*. 2016 (4)
TY0733	O157:H7 Δ*per*::*cat*	Washizaki *et al*. 2016 (4)
TY0750	O157:H7 Δ*ompC*::*kan*	Washizaki *et al*. 2016 (4)
TY0751	O157:H7 Δ*waaI*::*cat* Δ*ompC*::*kan*	Washizaki *et al*. 2016 (4)
TY0752	O157:H7 Δ*waaJ*::*cat* Δ*ompC*::*kan*	Washizaki *et al*. 2016 (4)
BB	B strain wild-type *sup^0*	Kai *et al*. 1999 (12)
B40su1	B strain *supD*	Kai *et al*. 1999 (12)
BL21(DE3)	F^- *ompT $hsdS_B(r_B^- m_B^-)$ gal(λcI 857, ind1, Sam7, nin5, lacUV5-T7gene1) dcm(DE3)*	Studier and Moffatt, 1986 (13)

TABLE S2 Oligonucleotides used in this study

**Primer name**	**Sequence (5′– 3′)**
T2g38-1up	GTCGACAAGAGGGCTTCGGCCCTC
T2g38-dw	AAGCTTTTAGACTCTTGAGCCGTAAA
T2g37-1up	AAGCTTGGCGAGGAGCGTCCGGTT
T2g37-2dw	TTACATAAGTTTAGATTCAAG
T2t-2up	CTTGAATCTAAACTTATGTAATTTTTGAATAAATATCTTA
T2t-1dw	GTCGACATGAAGGTAGAAAGTTAAAT
YO-763	CAGGTTTTTACCTTTAGACTGCAGG

172	YO-764	GCTGTCATCAATGGTCGTAACTACGACG
173	YO-765	GGTGCCATCAATGGTCGTAACTACGACG
174	YO-766	GGTGTCGCCAATGGTCGTAACTACGACG
175	YO-767	GGTGTCATCGCTAATGGTCGTAACTACG
176	YO-768	CCAGGTTTTTACCTTTAGACTGCAGG
177	YO-769	CTGTCATCAATGGTCGTAACTACGACG
178	YO-772	GGTGTCATCGCTGGTCGTAACTACG
179	YO-774	GGCTACGACGACGAAGATATCCTG
180	YO-775	ACGACCATTGATGACACCCAGGTTTTTAC
181	YO-782	GGTGTCATCGGTCGTAACTACGACG
182	YO-783	GTCATCAATGGTCGTAACTACGACG
183	YO-796	GGTGTCATCAATGGCGTAACTAACAAC
184	YO-797	TTCACCAGATGGGTTGCCGTTTTTAC
185	YO-821	CTGTACATCGGTAACGGCGACCGTG
186	YO-822	GCTGTTCTGATCATCAGTACGTTTG
187	YO-823	GACCTGTACATCGGTAACGGCGACC
188	YO-910	GGCAGCCAGGTTTTTACCTTTAGAC
189	YO-911	GCCGCTGGTCGTAACTACGACGACG
190	YO-933	GGTATCAATGGTCGTAACTACGACG
191	YO-934	GGTGTCAATGGTCGTAACTACGACG
192	YO-935	GGTCCCATCAATGGTCGTAACTACG
193	YO-936	GGTGTCCCCAATGGTCGTAACTACG
194	YO-937	GGTGTCATCCCTGGTCGTAACTACG
195	YO-940	GCCATCGGTAACGGCGACCGTGCTG
196	YO-941	CAGCGGGCTGTTCTGATCATCAGTA

197	YO-942	GCCGACGACGAAGATATCCTGAAAT
198	YO-943	GTTACGACCATTGATGACACCCAGG
199	YO-949	GCGGTACCCGCAGTAGCAGGACCGTGGATAGG
200	YO-950	GCCTGCAGTTATACTCTTGAACCATATATAGC
201	YO-988	GGACGTGGCGGTAATGGTTGGGCCG
202	YO-989	GGTGAATTCCTCCTGCTAGCCC
203	YO-990	CAGGAGGAATTCACCATGGCAATTGTAGGTGTTCC
204	YO-991	CATTACCGCCACGTCCATAAACAGTCACACCGCTATTAATG
205	YO-996	GCTTCAACACCTCCGACAAATCCTG
206	YO-997	ACCAGCACCTGGATTAGCATATTGT
207	YO-998	TCAACACCTCCGACAAATCCTGGTG
208	YO-999	GCGTATTATGCAGGTGGCGGTGGTG
209	YO-1000	TGTACCAGTATTACCGCCAGCACCC
210	YO-1001	TATTATGCAGGTGGCGGTGGTGAAG
211	YO-1002	TGTAGTACCACCTGTACCGGCATCTG
212	YO-1003	AGCTTGGCTGTTTTGGCGGATGAGAG
213	YO-1004	TACAGGTGGTACTACAACAGGGTATTATGCAGGTGGC
214	YO-1005	CCAAAACAGCCAAGCTTTATACTCTTGAACCATATATAG
215	YO-1006	ATTGATGACACCCAGGTTTTTACCTTTAGACTGC
216	YO-1007	GGTCGTGGCTACGACGACGAAGATAT
217	YO-1008	CCGGTACCTAAAAAAGCAAATAAAGGCA
218	YO-1009	CCAAGCTTTGTACGCTGAAAACAATG
219	YO-1010	AACCCATCTGGTGAAGGCGTAACTAACAAC
220	YO-1011	GTTGTTAGTTACGCCTTCACCAGATGGGTT
221	YO-1012	CCTTCAACACCTCCGACAAATC

222	YO-1026	GGAGCTATTGCCGGTGGCGG
223	YO-1027	CAGGAGGAATTCACCATGGCAATTGTAGGTGTTCC
224	YO-1028	CACCGGCAATAGCTCCGTTATTACGAATACGGAG
225	YO-1038	TGTGGCGGCGGTGGCGGTCG
226	YO-1039	GCCACCGCCGCCACAGCTACCATTAGTTGGATAGC
227	YO-1090	CGAATTAATAATGGCGGCGCTATTGCCGGTGGTGG
228	YO-1091	GCCATTATTAATTCGCAGAC
229	YO-1178	GCTTCACAAGCCAACAACTGGGC
230	YO-1179	TATTCACTAGCCAACAACTGGGC
231	YO-1180	ACAAAATATGTTTGTGGCGGCGG
232	YO-1181	CGCCCAGTTGTTGGCTTGTG
233	YO-1182	GGAAACTATGTTTGTGGCGGCGG
234	YO-1183	GGAAAAGGTGTTTGTGGCGGCGG
235	YO-1184	GGAAAATATAGTTGTGGCGGCGG
236	YO-1193	GCTTATTCACAAGCCAACAACTG
237	YO-1194	ACCGCCGCCACCGCCGCCAC
238	YO-1195	AAGAACAACTGGGCGGGAAAATATG
239	YO-1196	TTGTGAATAACCACCGCCGC
240	YO-1197	GCCAACTCCTGGGCGGGAAAATATG
241	YO-1198	GCCAACAACTACGCGGGAAAATATG
242	YO-1199	GCCAACAACTGGCCGGGAAAATATG
243	pp01-g38G41-EcoRI(+)	TATATGAATTCGGGTCGATCTGTTGAAGTAATTC
244	pp01-nonSTP-Hind3(-)	AATTAAGCTTTGATACTCTTGAACCATATATAGC
245	pp01-STP-Hind3(-)	AATTAAGCTTATACTCTTGAACCATATATAGCACC
246	PP01gp38_155_inv(-)	GCCACCGCCGCCACCGGCAATAG

PP01_gp38_Y159am_inv(+)	GGCGGTGGTTAGTCACAAGCCAACAACTGGGCGGG
PP01gp38_162_inv(-)	GGCTTGTGAATAACCACCGCCGCCACCG
PP01_gp38_Q161am_inv(+)	GGCGGTGGTTATTCATAGGCCAACAACTGGG
PP01_gp38_N164am_inv(+)	AACTAGTGGGCGGGAAAATATGTTTGTGGCGGCG
PP01_gp38_A166am_inv(+)	AACAACTGGTAGGGAAAATATGTTTGTGGCGGCG
PP01_gp38_Y169am_inv(+)	AACAACTGGGCGGGAAAATAGGTTTGTGGCGGCG
PP01gp38_206_inv(-)	ACCAGTATTACCGCCAGCACCCGGAGACGTC
PP01_gp38_T207am_inv(+)	TAGGGGTATTATGCAGGTGGCGGTGGTGAAGTTG
PP01_gp38_G208am_inv(+)	ACATAGTATTATGCAGGTGGCGGTGGTGAAGTTG
PP01_gp38_Y209am_inv(+)	ACAGGGTAGTATGCAGGTGGCGGTGGTGAAGTTG
PP01gp38_121_inv(-)	AGCGGCCCAACCATTACCGCC
PP01_gp38_G122am_inv(+)	TAGGCAATTGGAGCATCTGATGGCGGTGTATG
PP01_gp38_A123am_inv(+)	GGTTAGATTGGAGCATCTGATGGCGGTGTATG
PP01_gp38_I124am_inv(+)	GGTGCATAGGGAGCATCTGATGGCGGTGTATGTATCC
PP01gp38_185_inv(-)	ATTATTTCCGCCTAAGCCGAATGGACG
PP01_gp38_G186am_inv(+)	TAGGCTCGATGGCCTGGTGGTAATGCTAG
PP01_gp38_R188am_inv(+)	GGTGCTTAGTGGCCTGGTGGTAATGCTAGTTTGACG
PP01_gp38_W189am_inv(+)	GGTGCTCGATAGCCTGGTGGTAATGCTAGTTTGACG
PP01gp38_226_inv(-)	TGGATTAGCATATTGTCCTGGCTGACCAAC
PP01_gp38_G227am_inv(+)	TAGGCTGGTTATTCAACACCTCCGACAAATCCTG
PP01_gp38_A228am_inv(+)	GGTTAGGGTTATTCAACACCTCCGACAAATCCTGGTG
PP01_gp38_G229am_inv(+)	GGTGCTTAGTATTCAACACCTCCGACAAATCCTGGTGCTG
PP01_gp38_Y230am_inv(+)	GGTGCTGGTTAGTCAACACCTCCGACAAATCCTG

TABLE S3 Primers and template DNA used for mutagenesis (Inverse PCR)

Plasmids	Primers	Template plasmids
pBAD33-OmpC$_{K12\Delta GFTS+O157GVIN(L4)}$	YO-796 / YO-797	pBAD33-OmpC$_{K12\Delta GFTS}$
pBAD33-OmpC$_{K12\Delta GFTS+O157GVIN(L7)}$	YO-1006 / YO-1007	pBAD33-OmpC$_{K12\Delta GFTS}$
pBAD33-OmpC$_{O157(G303A)}$	YO-768 / YO-769	pBAD33-OmpC$_{O157}$ [Suga *et al.* 2021 (5)]
pBAD33-OmpC$_{O157(V304A)}$	YO-763 / YO-765	pBAD33-OmpC$_{O157}$
pBAD33-OmpC$_{O157(I305A)}$	YO-763 / YO-766	pBAD33-OmpC$_{O157}$
pBAD33-OmpC$_{O157(N306A)}$	YO-763 / YO-772	pBAD33-OmpC$_{O157}$
pBAD33-OmpC$_{O157(GVIN \rightarrow AAAA)}$	YO-910 / YO-911	pBAD33-OmpC$_{O157}$
pBAD33-OmpC$_{O157(N309G)}$	YO-774 / YO-775	pBAD33-OmpC$_{O157}$
pBAD33-OmpC$_{O157(\Delta G303)}$	YO-763 / YO-783	pBAD33-OmpC$_{O157}$
pBAD33-OmpC$_{O157(\Delta V304)}$	YO-763 / YO-933	pBAD33-OmpC$_{O157}$
pBAD33-OmpC$_{O157(\Delta I305)}$	YO-763 / YO-934	pBAD33-OmpC$_{O157}$
pBAD33-OmpC$_{O157(\Delta N306)}$	YO-763 / YO-782	pBAD33-OmpC$_{O157}$
pBAD33-OmpC$_{O157(V304P)}$	YO-763 / YO-935	pBAD33-OmpC$_{O157}$
pBAD33-OmpC$_{O157(I305P)}$	YO-763 / YO-936	pBAD33-OmpC$_{O157}$
pBAD33-OmpC$_{O157(N306P)}$	YO-763 / YO-937	pBAD33-OmpC$_{O157}$
pBAD33-OmpC$_{O157(Y228A)}$	YO-940 / YO-941	pBAD33-OmpC$_{O157}$
pBAD33-OmpC$_{O157(Y310A)}$	YO-942 / YO-943	pBAD33-OmpC$_{O157}$
pBAD33-OmpC$_{O157(P226D)}$	YO-822 / YO-823	pBAD33-OmpC$_{O157}$
pBAD33-OmpC$_{O157(\Delta P226)}$	YO-821 / YO-822	pBAD33-OmpC$_{O157}$
pBAD24-g38$_{T2-PP01(\Delta G208)}$	YO-1000 / YO-1001	pBAD24-g38$_{T2-PP01}$
pBAD24-g38$_{T2-PP01(G208A)}$	YO-999 / YO-1000	pBAD24-g38$_{T2-PP01}$
pBAD24-g38$_{T2-PP01(\Delta Y230)}$	YO-997 / YO-998	pBAD24-g38$_{T2-PP01}$
pBAD24-g38$_{T2-PP01(Y230A)}$	YO-996 / YO-997	pBAD24-g38$_{T2-PP01}$
pBAD24-g38$_{T2-PP01(Y230P)}$	YO-997 / YO-1012	pBAD24-g38$_{T2-PP01}$
pBAD24-g38$_{T2-PP01(G158A)}$	YO-1193/ YO-1194	pBAD24-g38$_{T2-PP01}$

pBAD24-g38$_{T2-PP01(Y159A)}$	YO-1171 / YO-1178	pBAD24-g38$_{T2-PP01}$
pBAD24-g38$_{T2-PP01(Q161L)}$	YO-1171 / YO-1179	pBAD24-g38$_{T2-PP01}$
pBAD24-g38$_{T2-PP01(A162K)}$	YO-1195 / YO-1196	pBAD24-g38$_{T2-PP01}$
pBAD24-g38$_{T2-PP01(N164S)}$	YO-1196 / YO-1197	pBAD24-g38$_{T2-PP01}$
pBAD24-g38$_{T2-PP01(W165Y)}$	YO-1196 / YO-1198	pBAD24-g38$_{T2-PP01}$
pBAD24-g38$_{T2-PP01(A166P)}$	YO-1196 / YO-1199	pBAD24-g38$_{T2-PP01}$
pBAD24-g38$_{T2-PP01(G167T)}$	YO-1180 / YO-1181	pBAD24-g38$_{T2-PP01}$
pBAD24-g38$_{T2-PP01(K168N)}$	YO-1181 / YO-1182	pBAD24-g38$_{T2-PP01}$
pBAD24-g38$_{T2-PP01(Y169G)}$	YO-1181/ YO-1183	pBAD24-g38$_{T2-PP01}$
pBAD24-g38$_{T2-PP01(V170S)}$	YO-1171 / YO-1184	pBAD24-g38$_{T2-PP01}$
his-PP01_gp38C-Y159am-pETDuet	PP01gp38_155_inv(-) / PP01_gp38_Y159am_inv(+)	his-PP01_gp38C - pETDuet
his-PP01_gp38C-Q161am-pETDuet	PP01gp38_162_inv(-) / PP01_gp38_Q161am_inv(+)	his-PP01_gp38C - pETDuet
his-PP01_gp38C-N163am-pETDuet	PP01gp38_162_inv(-) / PP01_gp38_N163am_inv(+)	his-PP01_gp38C - pETDuet
his-PP01_gp38C-N164am-pETDuet	PP01gp38_162_inv(-) / PP01_gp38_N164am_inv(+)	his-PP01_gp38C - pETDuet
his-PP01_gp38C-A166am-pETDuet	PP01gp38_162_inv(-) / PP01_gp38_A166am_inv(+)	his-PP01_gp38C - pETDuet
his-PP01_gp38C-Y169am-pETDuet	PP01gp38_162_inv(-) / PP01_gp38_Y169am_inv(+)	his-PP01_gp38C - pETDuet
his-PP01_gp38C-T207am-pETDuet	PP01gp38_206_inv(-) / PP01_gp38_T207am_inv(+)	his-PP01_gp38C - pETDuet
his-PP01_gp38C-G208am-pETDuet	PP01gp38_206_inv(-) / PP01_gp38_G208am_inv(+)	his-PP01_gp38C - pETDuet
his-PP01_gp38C-Y209am-pETDuet	PP01gp38_206_inv(-) / PP01_gp38_Y209am_inv(+)	his-PP01_gp38C - pETDuet
his-PP01_gp38C-G122am-pETDuet	PP01gp38_121_inv(-) / PP01_gp38_G122am_inv(+)	his-PP01_gp38C - pETDuet
his-PP01_gp38C-A123am-pETDuet	PP01gp38_121_inv(-) / PP01_gp38_A123am_inv(+)	his-PP01_gp38C - pETDuet
his-PP01_gp38C-I124am-pETDuet	PP01gp38_121_inv(-) / PP01_gp38_I124am_inv(+)	his-PP01_gp38C - pETDuet
his-PP01_gp38C-G186am-pETDuet	PP01gp38_185_inv(-) / PP01_gp38_G186am_inv(+)	his-PP01_gp38C - pETDuet
his-PP01_gp38C-R188am-pETDuet	PP01gp38_185_inv(-) / PP01_gp38_R188am_inv(+)	his-PP01_gp38C - pETDuet
his-PP01_gp38C-W189am-pETDuet	PP01gp38_185_inv(-) / PP01_gp38_W189am_inv(+)	his-PP01_gp38C - pETDuet
his-PP01_gp38C-G227am-pETDuet	PP01gp38_226_inv(-) / PP01_gp38_G227am_inv(+)	his-PP01_gp38C - pETDuet

his-PP01_gp38C-A228am-pETDuet	PP01gp38_226_inv(-) / PP01_gp38_A228am_inv(+)	his-PP01_gp38C - pETDuet
his-PP01_gp38C-G229am-pETDuet	PP01gp38_226_inv(-) / PP01_gp38_G229am_inv(+)	his-PP01_gp38C - pETDuet
his-PP01_gp38C-Y230am-pETDuet	PP01gp38_226_inv(-) / PP01_gp38_Y230am_inv(+)	his-PP01_gp38C - pETDuet

TABLE S4 Primers and template DNA used for mutagenesis (Gibson assembly)

**Plasmids**	**Insert : Template, Primers**	**Vector : Template, Primers**
pBAD24-g38$_{T2\text{-}PP01}$	pBAD28-g38$_{T2}$, YO-990 / YO-991	pBAD24-g38$_{PP01}$, YO-988 / YO-989
pBAD24-g38$_{T2\text{-}PP01(207\text{-}259)}$	pBAD24-g38$_{T2\text{-}PP01}$, YO-1004 / YO-1005	pBAD28-g38$_{T2}$, YO-1002 / YO-1003
pBAD24-g38$_{T2\text{-}PP01(158\text{-}259)}$	pBAD28-g38$_{T2}$, YO-1027/ YO-1028	pBAD24-g38$_{T2\text{-}PP01}$, YO-1026 / YO-989
pBAD24-g38$_{T2\text{-}PP01(171\text{-}259)}$	pBAD28-g38$_{T2}$, YO-1027 / YO-1039	pBAD24-g38$_{T2\text{-}PP01}$, YO-1038 / YO-989
pBAD24-g38$_{T2\text{-}PP01(113\text{-}157,\ 171\text{-}259)}$	pBAD28-g38$_{T2}$, YO-1039 / YO-1090	pBAD24-g38$_{T2\text{-}PP01}$, YO-1038 / YO-1091

REFERENCES FOR SUPPLEMENTAL MATERIALS

1. Doermann AH, Hill MB. 1953. Genetic structure of bacteriophage T4 as described by recombination studies of factors influencing plaque morphology. Genetics 38:79–90.

2. Kozloff LM, Lute M, Henderson K. 1957. Viral invasion. I. Rupture of thiol ester bonds in the bacteriophage tail. J Biol Chem 228:511–528.

3. Morita M, Tanji Y, Mizoguchi K, Akitsu T, Kijima N, Unno H. 2002. Characterization of a virulent bacteriophage specific forEscherichia coliO157:H7 and analysis of its cellular receptor and two tail fiber genes. FEMS Microbiol Lett 211:77–83.

4. Washizaki A, Yonesaki T, Otsuka Y. 2016. Characterization of the interactions between *Escherichia coli* receptors, LPS and OmpC, and bacteriophage T4 long tail fibers. Microbiologyopen 5:1003–1015.

5. Suga A, Kawaguchi M, Yonesaki T, Otsuka Y. 2021. Manipulating interactions between T4 phage long tail fibers and Escherichia coli receptors. Appl Environ Microbiol 87:e0042321.

6. Chin JW, Santoro SW, Martin AB, King DS, Wang L, Schultz PG. 2002. Addition of p-azido-L-phenylalanine to the genetic code of Escherichia coli. J Am Chem Soc 124:9026–9027.

7. Abramson J, Adler J, Dunger J, Evans R, Green T, Pritzel A, Ronneberger O, Willmore L, Ballard AJ, Bambrick J, Bodenstein SW, Evans DA, Hung C-C, O'Neill M, Reiman D, Tunyasuvunakool K, Wu Z, Žemgulytė A, Arvaniti E, Beattie C, Bertolli O, Bridgland A, Cherepanov A, Congreve M, Cowen-Rivers AI, Cowie A, Figurnov M, Fuchs FB, Gladman H, Jain R, Khan YA, Low CMR, Perlin K, Potapenko A, Savy P, Singh S, Stecula

A, Thillaisundaram A, Tong C, Yakneen S, Zhong ED, Zielinski M, Žídek A, Bapst V, Kohli P, Jaderberg M, Hassabis D, Jumper JM. 2024. Accurate structure prediction of biomolecular interactions with AlphaFold 3. Nature 630:493–500.

8. Discovery C, Boitreaud J, Dent J, McPartlon M, Meier J, Reis V, Rogozhnikov A, Wu K. 2024. Chai-1: Decoding the molecular interactions of life. bioRxiv.

9. Meng EC, Goddard TD, Pettersen EF, Couch GS, Pearson ZJ, Morris JH, Ferrin TE. 2023. UCSF ChimeraX: Tools for structure building and analysis. Protein Sci 32:e4792.

10. Madeira F, Madhusoodanan N, Lee J, Eusebi A, Niewielska A, Tivey ARN, Lopez R, Butcher S. 2024. The EMBL-EBI Job Dispatcher sequence analysis tools framework in 2024. Nucleic Acids Res 52:W521–W525.

11. Koga M, Otsuka Y, Lemire S, Yonesaki T. 2011. Escherichia coli rnlA and rnlB compose a novel toxin-antitoxin system. Genetics 187:123–130.

12. Kai T, Ueno H, Otsuka Y, Morimoto W, Yonesaki T. 1999. Gene 61.3 of bacteriophage T4 is the spackle gene. Virology 260:254–259.

13. Studier FW, Moffatt BA. 1986. Use of bacteriophage T7 RNA polymerase to direct selective high-level expression of cloned genes. J Mol Biol 189:113–130.
